# Assessing Polymer Biodegradability: Standardized Methods, Critical Challenges, and Future Directions

**DOI:** 10.3390/polym18172134

**Published:** 2026-09-01

**Authors:** Sarah Opinca-Tuef, Ioana Cristina Benea, Anamaria Todea, Ani Paloyan, Ioan Bîtcan, Francisc Péter

**Affiliations:** 1 Biocatalysis and Green Chemistry Group, Faculty of Chemical Engineering, Biotechnology and Environmental Protection, University Politehnica Timișoara, Vasile Pârvan 6, 300223 Timișoara, Romania; sarah.tuef@student.upt.ro (S.O.-T.); ioana.benea@upt.ro (I.C.B.); anamaria.todea@upt.ro (A.T.); francisc.peter@upt.ro (F.P.); 2 Scientific and Production Center “Armbiotechnology”, National Academy of Science of Armenia, Yerevan 0056, Armenia; anipaloyanm@gmail.com; 3 Research Institute for Renewable Energies (ICER), Politehnica University of Timisoara, Gavril Musicescu 138, 300501 Timişoara, Romania

**Keywords:** biodegradable polymers, biodegradation, standardized methods, ISO standards, ASTM standards, compostability, soil biodegradation, freshwater, marine biodegradation, environmental assessment

## Abstract

The rapid development of biodegradable polymers has intensified the need for reliable, standardized methodologies that can accurately assess their environmental fate. However, biodegradability is not an intrinsic property of materials, but rather the result of complex interactions between polymer chemistry, physicochemical characteristics, environmental conditions, and microbial activity. Consequently, biodegradation performance determined under one set of conditions cannot be directly extrapolated to other environments, highlighting the importance of selecting appropriate testing methodologies. This review provides a comprehensive and critical evaluation of the main international standards used to assess polymer biodegradability, including ISO, ASTM, EN, and OECD methods applicable to soil, industrial composting, freshwater, and marine environments. It discusses the fundamental mechanisms of polymer biodegradation, together with the major factors governing degradation kinetics, and comparatively analyzes the biodegradation behavior of polyesters, polysaccharides, polyamides, and polyesteramides across different environmental compartments. Unlike previous reviews, this work critically compares the applicability, strengths, limitations, and biodegradation endpoints of standardized methodologies, identifies current methodological gaps, and proposes a practical decision-making framework for selecting appropriate tests according to the intended end-of-life scenario of polymeric materials. Finally, it highlights the need for more harmonized and environmentally relevant testing approaches to better distinguish compostability from true environmental biodegradability.

## 1. Introduction

Biodegradability refers to the ability of materials to be chemically broken down by naturally occurring microorganisms (bacteria, fungi, and algae) into simpler compounds. The process is fundamentally an enzymatic depolymerization mechanism in which microorganisms use the material as a source of carbon and energy [1].

Biodegradable polymers have become increasingly important in efforts to address environmental challenges while still meeting industrial performance requirements. Their relevance is driven by the need to replace conventional plastics, which can persist for centuries and are a major source of global pollution [2]. The global packaging sector, dominated by petroleum-derived plastics, represents a primary target for biodegradable polymer adoption [2]. Biodegradable plastics excel in food service, agriculture, and perishable goods packaging, where environmental decomposition is beneficial. Biodegradable polymers address multiple agricultural needs while enhancing sustainability: films conserve moisture, increase soil temperature, reduce weeds, and can be left in the soil to biodegrade at the season’s end [3]. Polymers enable controlled delivery of pesticides, fertilizers, nutrients, and insect-repelling pheromones [3].

The biodegradable polymers considered in this review can be grouped into four distinct main classes, differentiated by their macromolecular architecture, biosynthetic or synthetic origin, and specific mechanisms of environmental or enzymatic degradation.

Aliphatic polyesters, particularly polylactic acid (PLA) and polyhydroxyalkanoates (PHAs), are the most widely studied biodegradable polyesters.

Polylactic acid offers favorable mechanical strength, biocompatibility, and non-toxic degradation products, making it suitable for packaging applications despite its relatively slow degradation rate (half-life ~168 days) [4]. Polyhydroxyalkanoates (PHAs) stand out as a particularly promising group of naturally occurring polyesters produced by a variety of microorganisms, typically under conditions of nutrient limitation. Owing to their tunable mechanical properties and their complete biodegradability, PHAs have been widely explored for use in areas such as packaging, biomedical devices, and agricultural films [4]. Their applicability extends further into specialty and consumer goods sectors; notably, PHAs have been utilized in printing toners and coating adhesives, while poly(3-hydroxybutyrate) (PHB), one of the most extensively studied PHA family members, has found use in small disposable items and packaging materials [3]. This broad application spectrum underscores the remarkable functional versatility of PHAs as next-generation biodegradable materials. The structures of both PLA and PHAs contain ester bonds that are prone to hydrolytic cleavage and enzymatic degradation by depolymerases. As a result, their degradation rates are strongly influenced by factors such as stereochemistry, degree of crystallinity, and environmental conditions, including temperature and humidity [5].

Polysaccharides, including cellulose and starch, are carbohydrate-based polymers with glycosidic linkages that undergo enzyme-driven degradation by cellulases and amylases, respectively. Their biodegradation is strongly influenced by structural characteristics, particularly the degree of crystallinity, polymerization, and accessibility of polymer chains to enzymatic attack [6]. Cellulose, the principal structural component of plant cell walls, consists of linear β-(1 → 4)-linked glucose units that form highly ordered crystalline structures, making it generally more resistant to biodegradation than starch. Its degradation is mediated by cellulolytic microorganisms, and the degradation rate depends on factors such as crystallinity, moisture, and environmental conditions [1,6].

Naturally derived polysaccharides, particularly starch, have attracted significant interest as sustainable alternatives to petroleum-based plastics. Starch is among the most widely available and economically accessible biodegradable polymers, commonly obtained from crops such as corn, rice, potatoes, and wheat. Compared with cellulose, its less ordered structure facilitates faster enzymatic hydrolysis and biodegradation under favourable environmental conditions [2]. Upon contact with soil microorganisms, extracellular amylases break down starch into low-molecular-weight fragments that are subsequently metabolized into carbon dioxide and water under aerobic conditions. Similar to cellulose, its degradation is influenced by crystallinity, hydrophilicity, temperature, humidity, and microbial activity [1,2]. Starch-based polymers are widely used in food packaging, disposable bags, agricultural mulch films, and other horticultural products designed to biodegrade at the end of their service life [3].

Polyamides, a family that includes both natural polyamino acids and synthetic nylons, feature amide backbones that tend to resist abiotic hydrolysis more stubbornly than ester bonds do. As a result, their breakdown relies more heavily on specific proteolytic or aminolytic enzymatic pathways rather than on simple chemical degradation [7,8].

To overcome the limited biodegradability of conventional polyamides while retaining their favourable mechanical properties, polyesteramides have emerged as a distinct class of heterochain polymers that strategically integrate amide and ester linkages within a single macromolecular backbone. This effectively enables bridging the gap between the high mechanical performance of polyamides and the inherent biodegradability of polyesters [9,10].

Across all these polymer classes, the rate and extent of biodegradation are shaped by a combination of factors: the polymer’s molecular structure, how it has been processed, and the environmental conditions it ultimately encounters. Understanding how these variables interact is not merely academic; it is central to choosing the right assessment methods in the first place [11].

Despite the growing interest in biodegradable polymers, accurately assessing their biodegradability remains a significant challenge. A central difficulty lies in the lack of standardized and universally applicable test methods, as current protocols leave considerable gaps in coverage across diverse environmental scenarios, including soil, compost, and aquatic systems [12].

Biodegradation rates are highly sensitive to environmental conditions such as temperature, humidity, and microbial community composition, meaning that a polymer deemed biodegradable under industrial composting conditions may persist largely intact in ambient environments [13]. Material-related factors further complicate the assessment of biodegradability, as properties such as crystallinity, surface area, molecular weight and processing history can significantly influence degradation rates [14]. Additionally, the formation of microplastic residues during incomplete degradation raises concerns about the true environmental impact of biodegradable plastics, highlighting the need for assessment methods that go beyond simple mass loss measurements [15]. These factors collectively underscore the need for more comprehensive, environment-specific methodologies and harmonized standards.

This review summarizes and critically compares the primary standardized methods used to evaluate the biodegradability of polymers, focusing on polyesters, polysaccharides, and polyamides (including polyesteramides). The main goal is to provide a comprehensive overview of the principles, applications, strengths, and limitations of current biodegradability assessment approaches, identifying the remaining gaps and challenges toward harmonized evaluation.

Although several review articles have summarized biodegradable polymers, their degradation mechanisms and the environmental performance of specific polymer families, comparatively few studies have critically evaluated the standardized methodologies used to assess polymer biodegradability in different environmental compartments. Furthermore, the differences in testing principles, measured endpoints, environmental relevance and the applicability of existing international standards are not discussed enough. This often leads to the incorrect assumption that biodegradability results obtained under one set of conditions can be directly extrapolated to other environments. This review aims to address these knowledge gaps by providing a comprehensive and critical comparison of internationally recognised biodegradability assessment standards (ISO, ASTM, EN and OECD) applicable to soil, industrial composting, freshwater and marine environments. In addition to summarising the biodegradation behaviour of the main classes of biodegradable polymers, this review evaluates the strengths, limitations, applicability and comparability of existing standardised methodologies. Furthermore, it distinguishes between compostability and environmental biodegradability, identifies current methodological limitations and proposes a practical framework to help select appropriate biodegradability tests according to the end-of-life scenario of polymeric materials. Thus, this review not only summarises current knowledge but also provides practical guidance for researchers, industry and regulatory stakeholders involved in developing and assessing the environmental impact of biodegradable polymers.

## 2. Fundamentals of Biodegradation

Polymer degradation occurs through both enzymatic and non-enzymatic (passive) mechanisms, which can frequently operate simultaneously in natural environments. Enzymatic processes involve bond-specific catalysis mediated by biological systems, whereas passive mechanisms such as hydrolysis, oxidation, photolysis, and thermal degradation modify polymer structures through physicochemical reactions that lack biological precision. Enzymatic activity is especially important for polymers that resemble natural substrates (amide- or glycosidic-containing backbones). Non-enzymatic degradation, also termed abiotic hydrolysis, occurs through chemical reactions with water molecules, particularly under conditions of elevated temperature or extreme pH. This mechanism is particularly relevant for polyesters such as PLA, where water molecules attack ester linkages, leading to chain scission and molecular weight reduction.

Environmental conditions dictate the final metabolic products: aerobic degradation, typical in composting and soil, mineralizing the polymer into carbon dioxide and water [16], while anaerobic degradation in oxygen-depleted environments, like landfills, leads to the production of methane and carbon dioxide [13].

### 2.1. Mechanisms of Biodegradation

**Figure 1:**
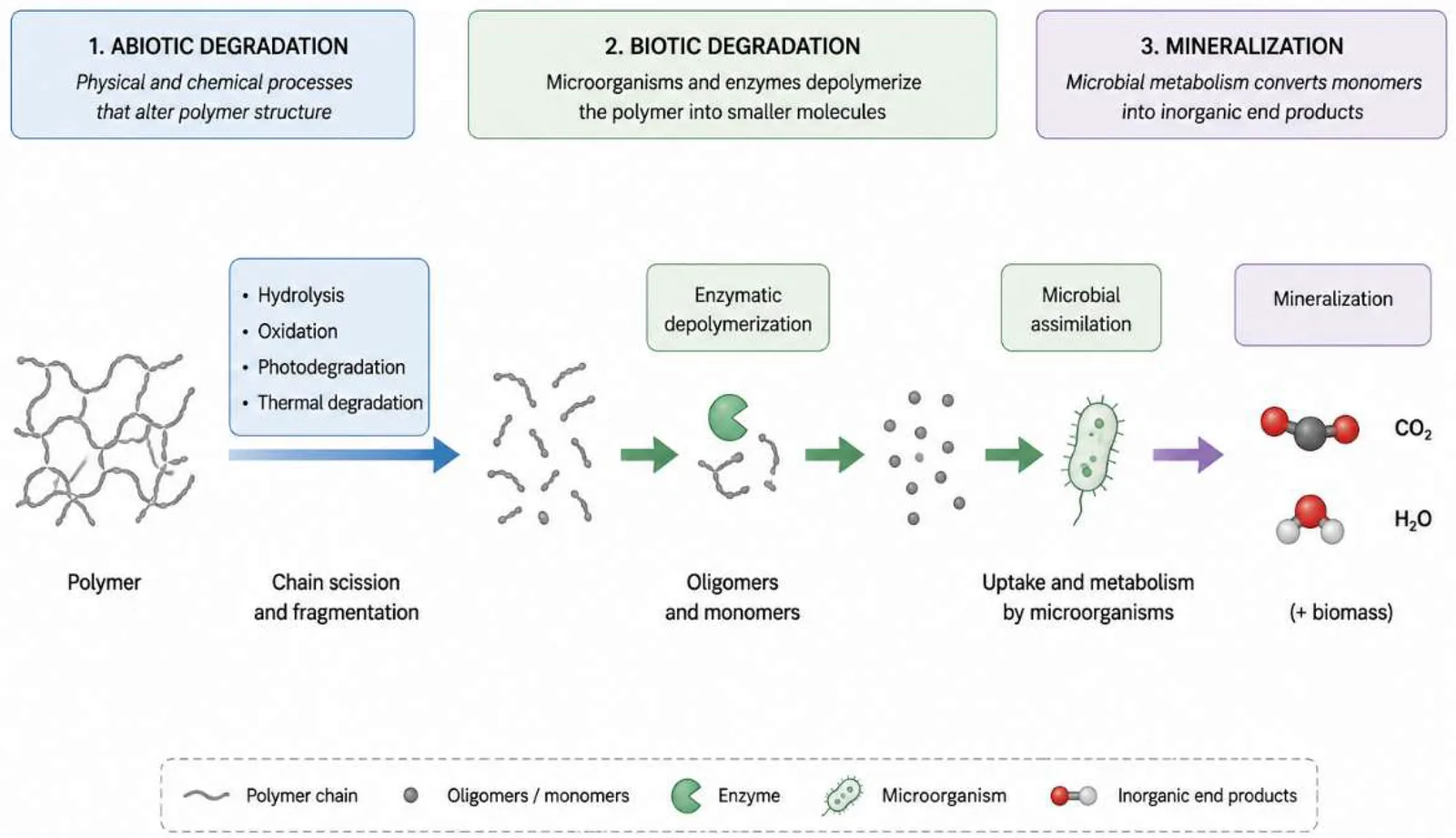
Fundamentals of polymer biodegradation: mechanisms and pathways.

Polymer biodegradation is a multistage process involving the sequential or simultaneous action of abiotic and biotic mechanisms that convert polymeric materials into inorganic compounds through microbial metabolism. Unlike simple physical fragmentation, true biodegradation requires the microbial assimilation of degradation products, followed by complete mineralization into carbon dioxide, water, methane (under anaerobic conditions), biomass, and mineral salts. Thus, biodegradation is governed by the interplay between polymer structure, environmental conditions, and microbial metabolic activity [1,16]. A schematic overview of the biodegradation mechanisms and pathways of polymers is shown in Figure 1.

#### 2.1.1. Abiotic Degradation

Abiotic degradation comprises non-biological, physicochemical processes that alter the polymer structure before or during biological degradation. The main mechanisms are hydrolysis, oxidation, photodegradation, and thermal degradation. These mechanisms contribute to chain scission and molecular weight reduction without direct microbial involvement [1,5].

Hydrolysis is the predominant abiotic pathway for condensation polymers containing hydrolyzable bonds, such as esters, polyesteramides, carbonates, urethanes, and anhydrides. Water molecules attack susceptible covalent bonds, generating oligomers and low-molecular-weight fragments that become more accessible to microbial enzymes. The hydrolysis rate depends strongly on temperature, moisture content, pH, crystallinity, and polymer morphology. Amorphous regions generally degrade more rapidly than crystalline domains due to their greater water permeability and chain mobility [5,17].

Oxidative degradation involves reactions between polymer chains and molecular oxygen or reactive oxygen species. These reactions lead to the formation of free radicals, carbonyl groups, hydroperoxides, and other oxygen-containing functionalities. These reactions progressively weaken the polymer backbone and facilitate subsequent enzymatic attack [1]. Similarly, exposure to ultraviolet radiation induces photodegradation by generating excited molecular states and radical species that are responsible for photooxidative chain cleavage. Thermal degradation contributes to polymer fragmentation by promoting bond cleavage at elevated temperatures, particularly under industrial composting conditions, where temperatures often exceed 55 °C [17].

While abiotic processes rarely achieve complete mineralization, they play a fundamental role by increasing surface roughness, reducing molecular weight, enhancing hydrophilicity, and exposing functional groups that promote microbial colonization and enzymatic accessibility [1,17].

#### 2.1.2. Biotic Degradation

Biotic degradation begins with the attachment of microorganisms to the polymer surface and the establishment of microbial biofilms. Surface colonization enables microorganisms to secrete extracellular depolymerizing enzymes that catalyze the cleavage of specific chemical bonds within the polymer backbone, generating soluble oligomers and monomers suitable for cellular uptake [1,6].

The enzymatic ensemble involved depends primarily on polymer chemistry. Polyesters are predominantly degraded by esterases, lipases, and cutinases, whereas polysaccharides are hydrolyzed by cellulases, amylases, chitinases, and chitosanases. In contrast, polyamides require amidases or proteases capable of cleaving the more hydrolytically stable amide bonds [6,11,18,19]. The efficiency of enzymatic depolymerization is influenced by several physicochemical properties, including crystallinity, molecular weight, chain flexibility, hydrophobicity, surface area, and degree of crosslinking, which collectively determines enzyme accessibility to susceptible bonds [5,17]. Once cleaved from the polymer matrix, oligomers and monomers are prone to be transported into microbial cells, where they enter central metabolic pathways such as glycolysis, the tricarboxylic acid cycle, or *β*-oxidation, depending on their chemical nature. These metabolic processes supply carbon and energy for microbial growth while progressively converting degradation products into inorganic compounds [16].

#### 2.1.3. Mineralization

Mineralization represents the final stage of polymer biodegradation and corresponds to the complete microbial conversion of organic carbon into inorganic end products. Under aerobic conditions, microorganisms oxidize degradation intermediates to carbon dioxide, water, biomass, and mineral salts. In anaerobic environments, such as landfills or anaerobic digesters, methanogenic microorganisms further convert metabolic intermediates into methane and carbon dioxide [13,16].

From the perspective of biodegradability assessment, mineralization constitutes the most reliable indicator of complete biodegradation because it demonstrates that polymer-derived carbon has been fully incorporated into natural biogeochemical cycles. Consequently, international standards such as ISO 14855-1 [20], ISO 14855-2 [21], ISO 17556 [22], ASTM D5338 [23], ASTM D5988 [24], ISO 18830 [25], and ISO 19679 [26] primarily quantify biodegradation through measurements of carbon dioxide evolution, oxygen consumption, or methane production, rather than relying solely on mass loss or physical disintegration, which may merely indicate fragmentation rather than true biodegradation [12,20,22].

Overall, polymer biodegradation is governed by the dynamic interaction between abiotic fragmentation, enzymatic depolymerization, microbial assimilation, and ultimate mineralization (Figure 1). The relative contribution of each mechanism depends on polymer chemistry, environmental conditions, and microbial community composition, explaining the substantial variability observed among different polymer classes and degradation environments.

Overall, the relative contribution of abiotic hydrolysis and enzymatic depolymerization varies considerably between different types of polymer. Polyesters generally degrade faster because their ester bonds are more susceptible to hydrolysis. In contrast, polyamides require more specialized enzymatic pathways due to the greater stability of amide bonds. Similarly, polysaccharides are typically degraded more efficiently as they are natural substrates for a wide range of microbial enzymes. These differences demonstrate that degradation efficiency depends not only on polymer chemistry but also on the interaction between material properties and environmental conditions.

It is also important to distinguish between the different endpoints used in biodegradation studies. Biodegradation refers to the overall microbial degradation of a polymer, whereas ultimate biodegradation is confirmed by mineralization—the conversion of polymer carbon into CO_2_, water, microbial biomass, and mineral salts under aerobic conditions, or into CO_2_ and CH_4_ under anaerobic conditions. Conversely, disintegration and mass loss represent physical changes to the material and should not be taken as evidence of complete biodegradation since fragmentation can occur without extensive microbial mineralization. Consequently, the endpoint measured by each standardized method must be carefully considered when interpreting and comparing biodegradation results.

### 2.2. Factors Affecting Biodegradation

**Figure 2:**
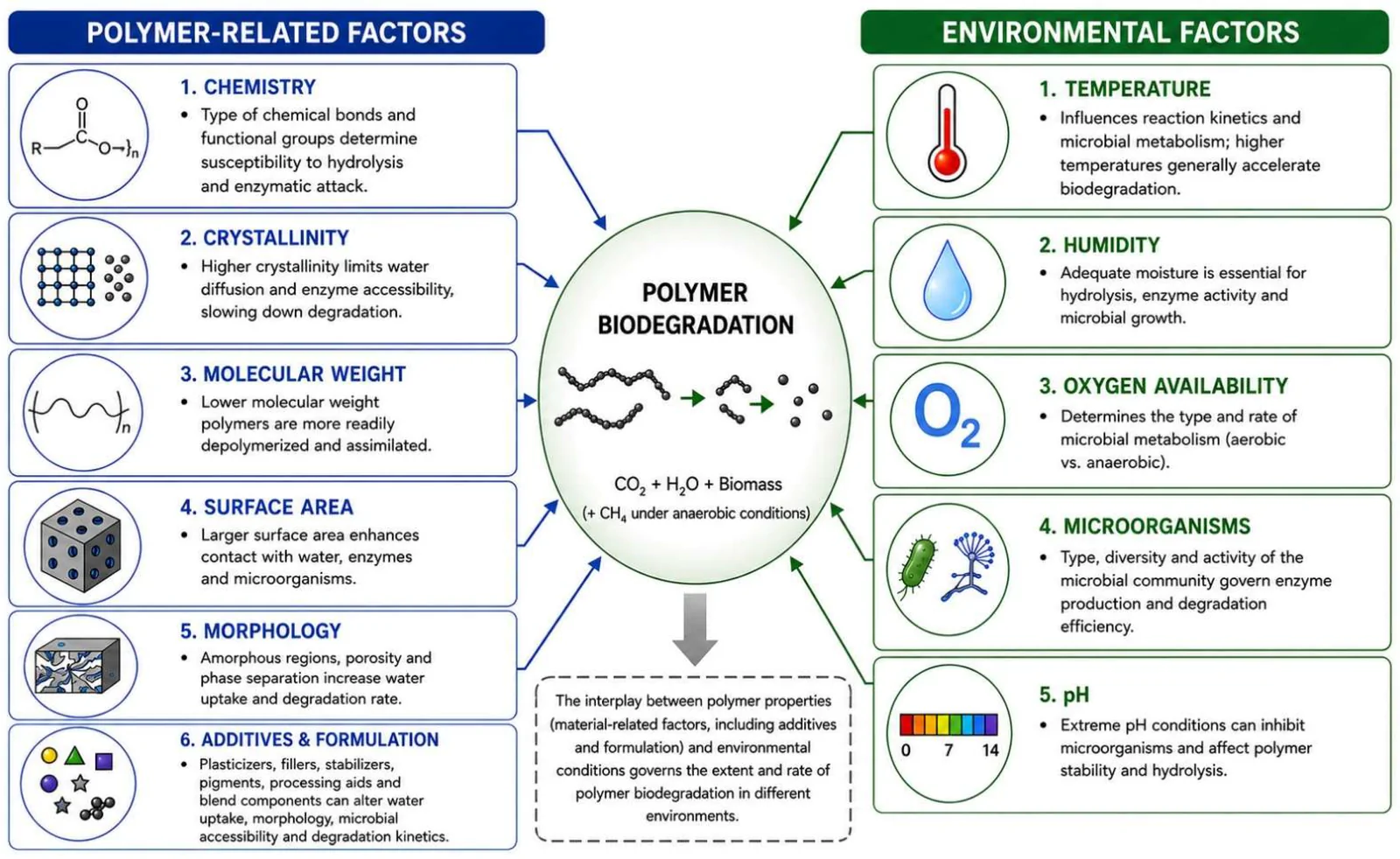
Factors influencing polymer degradation.

The biodegradation of polymers is linked to the intricate relationship between a material’s intrinsic physicochemical properties and the characteristics of its surroundings. Polymers with similar chemical structures may exhibit different degradation rates depending on factors such as molecular architecture, crystallinity, environmental conditions, and microbial activity (Figure 2). Therefore, biodegradation should be considered a material–environment interaction rather than an inherent polymer property [1,3].

#### 2.2.1. Polymer-Related Factors

The primary factor contributing to a polymer’s susceptibility to biodegradation is its chemical structure. Biodegradable polymers containing hydrolyzable functional groups, including PLA, PCL, PBS, PHAs, polyamides, and polyesteramides, generally degrade more readily than polymers with chemically stable carbon–carbon backbones or highly aromatic structures. The presence, distribution, and accessibility of these functional groups determine the affinity of hydrolytic enzymes toward the polymer backbone and strongly influence depolymerization kinetics [1,4,5].

The composition of the polymeric material should also be considered, as commercial and application-specific materials may contain plasticizers, fillers, compatibilizers, or other additives that can modify biodegradation behaviour. These components may alter the accessibility, hydrophilicity, morphology, and microbial interactions of the polymer matrix, thereby affecting degradation kinetics [27,28,29]. For example, plasticizers have been shown to influence PLA biodegradation under composting conditions, while natural fillers can modify the biodegradation behaviour of PHBV-based composites [27,29]. Similarly, the biodegradation of plasticized and blended cellulose-based materials may differ from that of the corresponding unmodified material [28]. Therefore, biodegradability should be considered at the level of the formulated polymeric material and not solely on the basis of the neat polymer.

Crystallinity governs the accessibility of water molecules and extracellular enzymes to polymer chains. In polymers such as PLA, PCL, PHAs, and cellulose, highly crystalline regions restrict water diffusion and enzyme penetration, thereby slowing hydrolysis and microbial degradation. In contrast, amorphous regions exhibit greater chain mobility and free volume, making them more susceptible to enzymatic attack. Consequently, biodegradation generally initiates within amorphous domains before progressively extending into crystalline regions [1,2,6].

Molecular weight further affects biodegradation. High-molecular-weight polymers, such as PLA and PCL, are less readily assimilated because extracellular enzymes first cleave polymer chains into smaller oligomers before microbial uptake can occur. As degradation progresses, chain scission reduces molecular weight, increases chain mobility, and accelerates further enzymatic depolymerization, creating a positive feedback mechanism that enhances degradation rates [2,6].

Surface area is equally important because biodegradation predominantly occurs at the polymer–environment interface. Polymers with larger specific surface areas, such as electrospun fibers, porous scaffolds, and finely divided particles, have greater contact with the polymer, water, microorganisms, and extracellular enzymes [6,11].

Polymer morphology, including porosity, surface roughness, molecular orientation, and phase distribution, also influences biodegradation by affecting water uptake, microbial adhesion, and enzyme accessibility. For example, the morphology of PLA/PBAT blends and starch-based composites can significantly influence degradation behaviour by altering water diffusion and microbial colonization. Likewise, increased surface roughness promotes biofilm formation, while porous structures facilitate the transport of water and degradation products throughout the polymer matrix [5,11].

#### 2.2.2. Environmental Factors

Environmental conditions strongly influence abiotic and biotic degradation mechanisms. Temperature is one of the most influential variables because it affects hydrolysis kinetics, enzyme activity, and microbial metabolism. Generally, elevated temperatures accelerate biodegradation by increasing molecular mobility and enhancing enzymatic activity. This explains why degradation is considerably faster under industrial composting conditions than in soil or marine environments [4,12].

Water participates directly in the hydrolysis of susceptible chemical bonds while simultaneously supporting microbial colonization and enzyme diffusion. Insufficient humidity limits abiotic hydrolysis and microbial activity, resulting in substantially slower biodegradation rates [1,2].

Oxygen availability determines the dominant microbial metabolic pathways. Under aerobic conditions, microorganisms oxidize degradation products to carbon dioxide, water, and biomass. In contrast, anaerobic environments promote methane production through methanogenic pathways. Consequently, the kinetics of biodegradation and the final degradation products differ substantially between aerobic composting systems and anaerobic digesters or landfill environments [4,13].

The composition and activity of microbial communities also play a decisive role in polymer biodegradation. Bacteria and fungi produce extracellular enzymes with different substrate specificities, and their abundance varies considerably among soil, freshwater, marine, and compost ecosystems. Therefore, environmental biodiversity determines not only the degradation rate but also the dominant enzymatic pathways involved in polymeric material depolymerization [2,12].

Finally, pH affects both chemical hydrolysis and enzyme stability. Acidic or alkaline conditions can speed up the hydrolysis of vulnerable bonds. However, most microbial enzymes exhibit maximum catalytic activity within relatively narrow pH ranges. Deviations from these optimal conditions reduce enzymatic depolymerization efficiency, consequently decreasing biodegradation rates [1,5].

Overall, the literature consistently shows that polymer chemistry and crystallinity are the main intrinsic factors that govern biodegradation, while temperature and microbial activity are the main environmental factors that influence it. While high temperatures and amorphous polymer structures generally accelerate biodegradation, the relative importance of each factor varies depending on the polymer family and testing environment. This emphasises the need for standardised methodologies that account for these differences.

### 2.3. Biodegradation in Different Environments

Polymer biodegradation depends not only on the intrinsic properties of the degraded material but also on the environment in which the degradation occurs. Although the overall pathway generally involves abiotic fragmentation, enzymatic depolymerization, microbial assimilation, and mineralization, the efficiency of each step varies markedly across soil, compost, freshwater, and marine systems. Temperature, moisture, oxygen availability, salinity, nutrient levels, and microbial diversity collectively determine the final rate and extent of biodegradation. Therefore, biodegradability should be regarded as an environment-specific property rather than an intrinsic and universally transferable material characteristic [1,2,3].

#### 2.3.1. Soil

Soil is one of the most relevant environmental matrices for evaluating polymer biodegradation because many agricultural, packaging, and consumer products ultimately enter terrestrial ecosystems once they reach the end of their service life. Soil is a highly heterogeneous environment, unlike controlled laboratory conditions. It is characterized by fluctuations in temperature, moisture, oxygen availability, pH, nutrient content, and microbial diversity. These variables directly influence microbial metabolism, extracellular enzyme production, and hydrolytic reactions. Consequently, biodegradation rates vary considerably among different geographical locations and soil types. Consequently, soil biodegradation tests are generally considered more representative of real environmental conditions than industrial composting, though they are less reproducible due to environmental heterogeneity [1,30,31].

Among biodegradable polymers, polyesters generally exhibit the highest biodegradation rates in soil due to the hydrolyzable nature of ester bonds. Their degradation proceeds through a combination of abiotic hydrolysis and enzymatic depolymerization, which is primarily catalyzed by extracellular esterases, lipases, and cutinases that are secreted by soil microorganisms. Initial hydrolysis reduces the polymer’s molecular weight, generating oligomers and monomers that can be assimilated and metabolized by bacteria and fungi [32]. The efficiency of this process depends strongly on polymer morphology and physicochemical properties. Materials with larger specific surface areas, such as electrospun fibers, degrade considerably faster than compact films because they provide greater enzyme accessibility and facilitate microbial colonization [14].

Polyhydroxybutyrate (PHB) and poly(butylene adipate-co-terephthalate) (PBAT) blends illustrate the influence of polymer structure well. Under mesophilic soil conditions, PHB/PBAT bilayer films achieved approximately 47% mineralization within 180 days, whereas PHB underwent nearly complete degradation. PBAT became progressively more crystalline during incubation, demonstrating that biodegradation occurs first in amorphous regions and then extends into crystalline domains [30]. Similar observations have been reported for poly(lactic acid) (PLA), which hydrolyzes relatively slowly under ambient temperatures, limiting its degradation in soil compared to PHAs or aliphatic PBS-based materials [31].

Polyamide polymers display considerably greater resistance to biodegradation than polyesters because amide bonds are chemically more stable and less susceptible to hydrolytic cleavage. Nevertheless, recent studies have demonstrated that certain microorganisms can degrade selected aliphatic polyamides. For instance, *Brevibacillus brevis*, which was isolated from soil, has been reported to degrade micro-sized nylon 6,6 particles. Additionally, polyamide 4 (PA4) undergoes enzymatic degradation through the action of amidases and proteases produced by soil microorganisms, particularly strain NR4. Despite these advances, the biodegradation of conventional nylons remains substantially slower than that of biodegradable aliphatic polyesters.

Polysaccharides are the most biodegradable polymer family in terrestrial environments because they are naturally occurring substrates in the global carbon cycle. Starch is rapidly hydrolyzed by microbial amylases into oligosaccharides and glucose. In contrast, cellulose degradation is catalyzed by cellulases, which can cleave β-(1→4)-glycosidic bonds. The rate at which cellulose degrades is strongly influenced by its crystallinity; amorphous cellulose is considerably more susceptible to enzymatic attack than highly ordered crystalline structures. Similarly, chitin and chitosan are efficiently biodegraded by chitinases and chitosanases produced by fungi and actinomycetes, including Streptomyces species. Their natural occurrence in soil ecosystems explains their rapid mineralization relative to synthetic biodegradable polymers [33].

However, substantial differences remain among individual polymers due to variations in crystallinity, molecular weight, morphology, surface area, and microbial accessibility [1,14,31]. These findings underscore the fact that biodegradation cannot be predicted based solely on polymer chemistry; rather, it must be interpreted in the context of the surrounding soil ecosystem’s physicochemical characteristics.

#### 2.3.2. Industrial Composting

Industrial composting is the standard for evaluating the biodegradability of polymeric materials because it provides optimal conditions for microbial growth and enzymatic activity. Unlike natural environments, composting systems operate under controlled thermophilic conditions, typically at 58 ± 2 °C. These systems have a high moisture content, continuous aeration, and a dense population of microbes, including bacteria, fungi, and actinomycetes. These conditions accelerate abiotic hydrolysis and enzymatic depolymerization, resulting in the highest degradation rates of biodegradable polymers. Consequently, most international standards for assessing polymer biodegradability, including ISO 14855, ASTM D5338, EN 13432, and ASTM D6400, are based on industrial composting conditions [31,34,35].

Among biodegradable polymers, polyesters exhibit the most extensive degradation under composting conditions, owing to the hydrolyzable nature of ester linkages and the accelerated hydrolysis promoted by elevated temperatures. Water penetration into the polymer matrix initiates chain scission, producing soluble oligomers and monomers that are subsequently assimilated by compost microorganisms. This hydrolytic stage is followed by extracellular enzymatic degradation mediated primarily by esterases, lipases, and cutinases, resulting in complete mineralization into carbon dioxide, water, and microbial biomass [32].

The degradation behavior of polyesters varies considerably according to polymer chemistry. Poly(lactic acid) (PLA), despite exhibiting relatively slow degradation in soil, undergoes rapid hydrolysis under thermophilic composting conditions because temperatures above its glass transition temperature significantly increase chain mobility and water diffusion. Following hydrolysis, low-molecular-weight lactic acid oligomers are readily metabolized by compost microorganisms. Experimental studies have demonstrated progressive degradation of PLA/PBAT blends during compost storage, with weight losses increasing from approximately 7.4% after 30 days to nearly 30% after 90 days under controlled composting conditions [31]. Depending on compost maturity, temperature, and moisture content, complete mineralization of PLA-based materials may occur within approximately 90–180 days.

Other aliphatic polyesters, including poly(butylene succinate) (PBS), polyhydroxyalkanoates (PHAs), and poly(butylene adipate-co-terephthalate) (PBAT), typically degrade faster than PLA under mesophilic and thermophilic composting conditions. Comparative studies have shown that PBS and PBAT often begin to degrade earlier than PLA because their polymer chains are flexible enough to allow enzymes to access them at moderate temperatures. PHAs are naturally synthesized microbial polyesters that often display the highest biodegradation rates due to the widespread occurrence of PHA depolymerases in compost microbial communities [32]. Overall, composting provides nearly ideal conditions for polyester biodegradation and therefore represents the benchmark environment for evaluating biodegradable plastics.

Although polyamides are inherently more resistant to biodegradation due to the stability of amide bonds, composting substantially enhances their degradation compared to soil or aquatic environments. Elevated temperatures increase polymer chain mobility and stimulate microbial metabolism. The high enzymatic diversity in compost favors the proddouction of amidases and proteases that can cleave susceptible amide linkages [34].

Polysaccharides, such as starch, cellulose, chitin, and chitosan, generally undergo rapid biodegradation under composting conditions because they are naturally integrated into microbial metabolic pathways. Compost microorganisms produce a wide range of extracellular amylases, cellulases, chitinases, and chitosanases that efficiently hydrolyze glycosidic bonds, resulting in extensive mineralization within a relatively short period [1,2,36]. Native starch-based materials often degrade completely within a few weeks, whereas the biodegradation of cellulose is primarily governed by its supramolecular structure, particularly crystallinity, which influences the accessibility of cellulose chains to cellulolytic enzymes [9,36]. Although native cellulose is readily biodegradable, highly ordered crystalline regions can restrict enzymatic accessibility and consequently slow its biodegradation [9,36]. Importantly, chemical modification should not be considered a universal predictor of biodegradation rate. As discussed by Erdal and Hakkarainen [36], the biodegradability of cellulose derivatives depends on the nature of the chemical modification, the degree of substitution, and the resulting physicochemical properties. For example, carboxymethyl cellulose (CMC) remains readily biodegradable, demonstrating that chemical modification does not necessarily reduce biodegradability, whereas other cellulose derivatives, such as highly substituted cellulose acetate, may exhibit considerably slower biodegradation due to reduced enzymatic accessibility [31]. Therefore, the biodegradation behavior of cellulose derivatives should be evaluated individually according to their chemical structure and physicochemical properties rather than assuming that chemical modification universally reduces biodegradability relative to native cellulose [31].

An increasingly important class of biodegradable polymers is represented by polyesteramides (PEAs), which combine hydrolyzable ester bonds with mechanically robust amide segments. Their molecular architecture enables a balance between biodegradability and mechanical performance that cannot be achieved by conventional polyesters or polyamides alone. Under standardized composting conditions, several synthesized PEAs have demonstrated biodegradation levels exceeding 90% of the positive control within six months, thereby satisfying the compostability criteria established by the European Committee for Standardization (CEN) [37]. Importantly, ecotoxicological evaluation of the resulting compost revealed no adverse effects associated with PEA degradation products, confirming their suitability for compostable applications. The biodegradation rate of PEAs is largely governed by the ester-to-amide ratio; increasing ester content generally promotes faster hydrolysis while maintaining favorable mechanical properties.

Overall, composting is the optimal environment for evaluating polymer biodegradability because it combines elevated temperature, high moisture content, continuous aeration, and exceptional microbial diversity. However, the excellent degradation observed under industrial composting conditions should not be directly extrapolated to natural terrestrial or aquatic ecosystems. Materials that fulfill the requirements for composability according to international standards do not necessarily exhibit comparable biodegradation in soil, freshwater, or marine environments. This emphasizes the importance of selecting testing conditions that accurately reflect the intended end-of-life scenario [31,34,35].

#### 2.3.3. Freshwater

Freshwater ecosystems are important but comparatively understudied environments for assessing polymer biodegradation. Rivers, lakes, reservoirs, and wastewater systems are receiving increasing quantities of plastic waste from agriculture, packaging, textiles, and urban activities. This makes freshwater environments critical intermediate compartments between terrestrial and marine ecosystems. Unlike industrial composting, freshwater systems are characterized by low temperatures, diluted nutrient concentrations, variable dissolved oxygen levels, and strongly varying availability and activity of microorganisms. These factors reduce biodegradation kinetics. Furthermore, hydrodynamic conditions, seasonal fluctuations, and differences in microbial community composition introduce considerable variability among studies, complicating direct comparisons of biodegradation performance.

Among biodegradable polymers, polyesters are the most extensively studied family in freshwater environments. They degrade via a mechanism similar to that observed in terrestrial ecosystems: initial abiotic hydrolysis followed by microbial colonization, enzymatic depolymerization, and mineralization. However, because freshwater temperatures are generally below the glass transition temperature of most biodegradable polyesters, hydrolysis proceeds much more slowly than under composting conditions. Consequently, enzymatic degradation largely depends on the formation of microbial biofilms capable of maintaining prolonged contact between extracellular enzymes and the polymer surface [38].

Recent genome-centric metagenomic studies have demonstrated that freshwater plastisphere communities contain specialized bacterial consortia that can degrade biodegradable polyesters through coordinated metabolic pathways. Biofilm-forming microorganisms secrete extracellular esterases, lipases, and cutinases that hydrolyze ester bonds. The resulting oligomers are subsequently assimilated by microbial cells [38]. Environmental parameters, including dissolved oxygen concentration, nutrient availability, seasonal temperature fluctuations, and water flow velocity, significantly impact biofilm development and enzymatic activity. This results in considerable spatial and temporal variability in degradation rates [39].

Among commercial biodegradable polyesters, blends containing polyhydroxybutyrate (PHB), poly(lactic acid) (PLA), and poly(ε-caprolactone) (PCL) exhibit different degradation behaviors when in contact with freshwater. PHB generally has the highest biodegradation rate due to the prevalence of PHA depolymerases in aquatic microbial communities. In contrast, PLA degrades considerably more slowly due to limited hydrolysis at ambient temperatures. PCL exhibits intermediate behavior due to its relatively flexible polymer backbone and susceptibility to lipase-catalyzed hydrolysis [19]. Additionally, the biodegradation kinetics of polymer blends depend on polymer chemistry, crystallinity, phase morphology, and blend composition. This emphasizes the importance of physicochemical structure in determining freshwater degradation performance [40].

Compared with polyesters, polyamides are considerably more resistant to biodegradation in freshwater systems. Their hydrolytically stable amide bonds require specialized amidases and proteases, which are generally less abundant in aquatic microbial communities than ester-hydrolyzing enzymes. Consequently, conventional polyamides, such as PA6 and PA66, exhibit negligible degradation under normal freshwater conditions. The current literature contains only a few reports describing the biodegradation of specific aliphatic polyamides, indicating that the biodegradation of this polymer family in freshwater remains poorly understood and requires further investigation [41].

Polysaccharides are substantially more biodegradable than synthetic polymers in freshwater because aquatic microorganisms naturally recognize them. Experimental studies using biochemical oxygen demand (BOD) measurements have reported aerobic biodegradation values of approximately 30–50% after 30 days. Weight-loss analyses have demonstrated 50–80% mass reduction over the same period. These results suggest that enzymatic hydrolysis and microbial mineralization occur efficiently in freshwater ecosystems, even at relatively low temperatures. Similarly, starch-based materials undergo rapid enzymatic degradation through the action of extracellular amylases. Chitin and chitosan, on the other hand, are biodegraded by chitinases and chitosanases produced by aquatic bacteria and fungi [36,42]. Their natural occurrence in the carbon cycle of freshwater ecosystems explains their consistently high biodegradation rates.

The biodegradation of polyesteramides (PEAs) in freshwater environments remains largely unexplored. Very few experimental studies have directly evaluated PEA degradation in rivers or lakes, creating a significant knowledge gap in the current literature. Therefore, existing evidence is largely indirect and based on the biodegradation behavior of structurally related polyesters and polyamides. Since PEAs contain both hydrolyzable ester bonds and more stable amide linkages, their biodegradation is expected to fall between that of these two polymer families. However, the relative contribution of hydrolysis and enzymatic cleavage depends heavily on polymer composition, the ester-to-amide ratio, molecular architecture, and microbial community composition [37,41]. The scarcity of standardized freshwater studies underscores the necessity of further research to address the environmental fate of this emerging class of biodegradable polymers.

Compared with soil and compost, biodegradation in freshwater generally proceeds at considerably lower rates due to reduced temperatures and lower microbial activity. However, freshwater ecosystems more realistically represent the environmental exposure experienced by numerous packaging materials, fibers, and consumer products before their eventual transport into marine environments. Consequently, freshwater testing plays an increasingly important role in environmental risk assessment, complementing conventional compostability evaluations.

Despite growing interest in freshwater biodegradation, standardized methodologies are relatively limited compared to those for soil or compost. The lack of harmonized protocols, together with the high variability of natural aquatic environments, continues to hinder direct comparisons among published studies. Therefore, developing robust freshwater biodegradability standards is one of the most important future directions for improving the environmental evaluation of biodegradable polymers.

#### 2.3.4. Seawater

Marine ecosystems are the most challenging environments for polymer biodegradation. They combine unfavorable physicochemical and biological conditions that limit abiotic and biotic degradation processes. Compared with terrestrial ecosystems, seawater is characterized by lower temperatures, high salinity, lower nutrient availability, reduced microbial biomass, and distinct microbial communities. These factors decrease hydrolysis rates, enzymatic activity, and microbial metabolism. Consequently, polymers that readily biodegrade under industrial composting conditions often degrade only minimally in marine environments. This emphasizes the importance of evaluating biodegradable materials under application-specific environmental conditions rather than extrapolating compostability data to aquatic ecosystems [43,44,45].

Polyesters are the most extensively studied biodegradable polymers in marine environments due to their widespread use in packaging, fishing gear, agricultural products, and single-use applications that often end up in coastal and offshore ecosystems. Their degradation mechanism is similar to that observed in soil and freshwater environments and involves abiotic hydrolysis, microbial colonization, extracellular enzymatic depolymerization, and mineralization. However, low seawater temperatures substantially reduce hydrolysis kinetics. High salinity and nutrient limitation further restrict microbial activity, resulting in considerably slower degradation than under composting conditions.

Recent studies have demonstrated the biodegradation of poly(butylene succinate) (PBS) fishing gear buried in marine sediments. These studies confirm that biodegradable polyesters can degrade in seawater, though the process requires a significantly longer incubation period than in soil or compost. Comparative investigations involving PLA, PBS, and PBAT have demonstrated that biodegradation rates vary among polyester families, reflecting differences in polymer crystallinity, chain flexibility, and susceptibility to enzymatic hydrolysis. Similarly, PHB/PLA/PCL blends exhibit degradation patterns that are strongly influenced by polymer composition. PHB generally degrades faster than PLA or PCL due to the prevalence of PHA-degrading microorganisms in marine biofilms [46].

Compared to biodegradable polyesters, polyamides are considerably more resistant to marine biodegradation because amide bonds are more stable and require specialized enzymes to break down. Consequently, conventional nylons persist for extended periods in marine environments. However, measurable degradation of polyamide 6 (PA6) fibers has been reported, showing that marine microorganisms can produce amidases and proteases that gradually cleave amide linkages. While these results show the potential for the biodegradation of certain aliphatic polyamides, their degradation rates are substantially lower than those of aliphatic polyesters.

In contrast, polysaccharides exhibit excellent biodegradability because they participate in marine carbon cycling. Chitin, one of the most abundant marine biopolymers, is easily broken down by marine bacteria and fungi that produce highly efficient chitinases. Cellulose also undergoes microbial degradation in seawater, but its mineralization is slower than that in freshwater environments. Biochemical oxygen demand (BOD) measurements indicate only 2–10% aerobic mineralization after 30 days, whereas weight-loss measurements reveal considerably greater mass losses (approximately 60–80%) during the same period [42]. This apparent discrepancy illustrates the important distinction between physical disintegration and complete biodegradation. Although cellulose fibers may fragment extensively under marine conditions, complete microbial mineralization into carbon dioxide and water occurs much more slowly due to the limited microbial activity characteristic of marine ecosystems.

An emerging strategy for enhancing marine biodegradation is the development of polyesteramides (PEAs), which are designed specifically for aquatic applications. Unlike conventional biodegradable polyesters, PEAs have hydrolyzable ester bonds and amide segments, which enhance mechanical performance and microbial interactions. Recent studies have demonstrated that incorporating amide functionalities can facilitate microbial colonization while maintaining adequate material durability during use. Sequential polyesteramides containing *γ*-aminobutyric acid units, in particular, exhibited significantly higher biodegradation in seawater than the corresponding homopolyesters after one month of incubation [47,48]. This indicates that rational molecular design can substantially improve degradation performance under marine conditions. These findings highlight PEAs as one of the most promising polymer families for future marine biodegradable materials.

Overall, however, marine biodegradation remains considerably slower than degradation in terrestrial environments. Temperature, salinity, nutrient limitation, oxygen availability, and microbial diversity collectively determine degradation kinetics, making marine biodegradability one of the most demanding criteria for biodegradable polymer design. It is important to note that recent research increasingly emphasizes that compostability should not be regarded as evidence of marine biodegradability since these environments differ fundamentally in physicochemical conditions and microbial ecology [45].

#### 2.3.5. Comparison of the Degradation Environments

A comparison of the four main degradation environments shows that biodegradation is influenced by the interaction between polymer chemistry and environmental conditions, rather than by polymer composition alone. Industrial composting provides the highest degradation rates because it maximizes both hydrolytic reactions and enzymatic depolymerization through elevated temperatures, high moisture content, continuous aeration, and abundant microbial populations. Soil environments exhibit lower, yet still substantial, biodegradation due to active microbial communities. However, degradation kinetics are strongly influenced by seasonal and geographical variability. Freshwater ecosystems generally support slower degradation due to lower temperatures and reduced microbial biomass. Marine environments are the least favorable due to low temperatures, high salinity, and nutrient limitation [31,38,43].

Across all environments, the considered polymer families exhibit a consistent biodegradation hierarchy:

Polysaccharides > polyesters > polyesteramides > polyamides.

Polysaccharides undergo rapid biodegradation because they are recognized by a broad range of microorganisms and extracellular enzymes. Polyesters are the largest family of synthetic biodegradable polymers and benefit from the presence of widespread microbial esterases, lipases, and cutinases that can hydrolyze ester bonds. Polyesteramides occupy an intermediate position, combining the biodegradability of polyesters with the superior mechanical properties of amide segments. Polyamides are the most degradation-resistant family due to the higher chemical stability of amide linkages [1,32,36,37].

The primary environments considered in this review, soil, industrial compost, freshwater, and marine ecosystems, substantially differ in microbial activity, environmental stability, and degradation kinetics, as summarized in Table 1.

As shown in Table 1, industrial composting provides the most favorable conditions for biodegradation. These conditions include elevated temperatures, high moisture content, efficient aeration, and intense microbial activity. These conditions significantly accelerate hydrolysis and enzymatic depolymerization, enabling many biodegradable polyesters to undergo extensive mineralization in just a few months. In contrast, soil environments are considerably more heterogeneous. Degradation rates in soil are strongly influenced by soil composition, seasonal temperature fluctuations, water availability, and microbial diversity. Consequently, biodegradation in soil generally occurs more slowly and is more variable than under controlled composting conditions [5,6,11,12].

Freshwater environments present additional limitations, including lower microbial biomass, reduced nutrient availability, and fluctuating physicochemical conditions. These factors contribute to slower degradation kinetics. Furthermore, freshwater biodegradation testing is less standardized than compost or soil testing, making comparisons between independent studies more challenging [13,14].

Marine environments are the most demanding scenario for biodegradable polymers. Low temperatures, high salinity, limited nutrient availability, and specialized microbial communities substantially reduce hydrolytic and enzymatic degradation. Consequently, polymers certified as compostable often undergo only partial degradation in marine environments, highlighting that compostability and marine biodegradability are not synonymous [14,15,16].

Overall, biodegradation generally follows the trend: Industrial Compost > Soil > Freshwater > Marine. This sequence reflects the progressive decline in environmental conditions favorable to hydrolysis, microbial growth, and enzymatic activity. Industrial composting provides the highest biodegradation efficiency owing to elevated temperature, controlled moisture, aeration, and high microbial activity, whereas marine environments represent the most challenging conditions because of lower temperatures, limited nutrient availability, and reduced microbial abundance. Soil and freshwater exhibit intermediate biodegradation rates, although considerable variability exists due to differences in environmental conditions and microbial communities.

These observations demonstrate that biodegradation performance is strongly environment-dependent and that results obtained in one environmental compartment cannot be directly extrapolated to another. Consequently, selecting an appropriate testing environment and standardized methodology is essential for accurately predicting the environmental fate of biodegradable polymers according to their intended end-of-life scenario. Despite significant progress in developing biodegradable polymers, further harmonization of international testing protocols remains necessary to improve the comparability and environmental relevance of biodegradability assessments.

## 3. Standard Methodologies for Assessing Biodegradation

### 3.1. Standard Methodologies for Assessing Plastic Biodegradation in Soil

International standards were developed primarily to provide standardized methodologies for product testing and certification. Their clearly defined procedures, controlled testing conditions, and reproducible evaluation criteria have led to their widespread adoption in scientific research for assessing the biodegradability of polymeric materials. Accordingly, this section reviews internationally recognized biodegradation test methods and discusses their application for evaluating biodegradable polymeric materials under different environmental conditions.

It is important to distinguish between standardized biodegradation test methods and product specification or certification standards. Biodegradation test methods (e.g., ISO 14855, ISO 17556, ASTM D5338, and ASTM D5988) describe standardized procedures for determining biodegradation under defined environmental conditions. In contrast, product specification and certification standards (e.g., EN 13432 and ASTM D6400) establish the requirements that materials or products must satisfy to be classified as compostable, including criteria for biodegradation, disintegration, ecotoxicity, and limits for chemical constituents.

**Figure 3:**
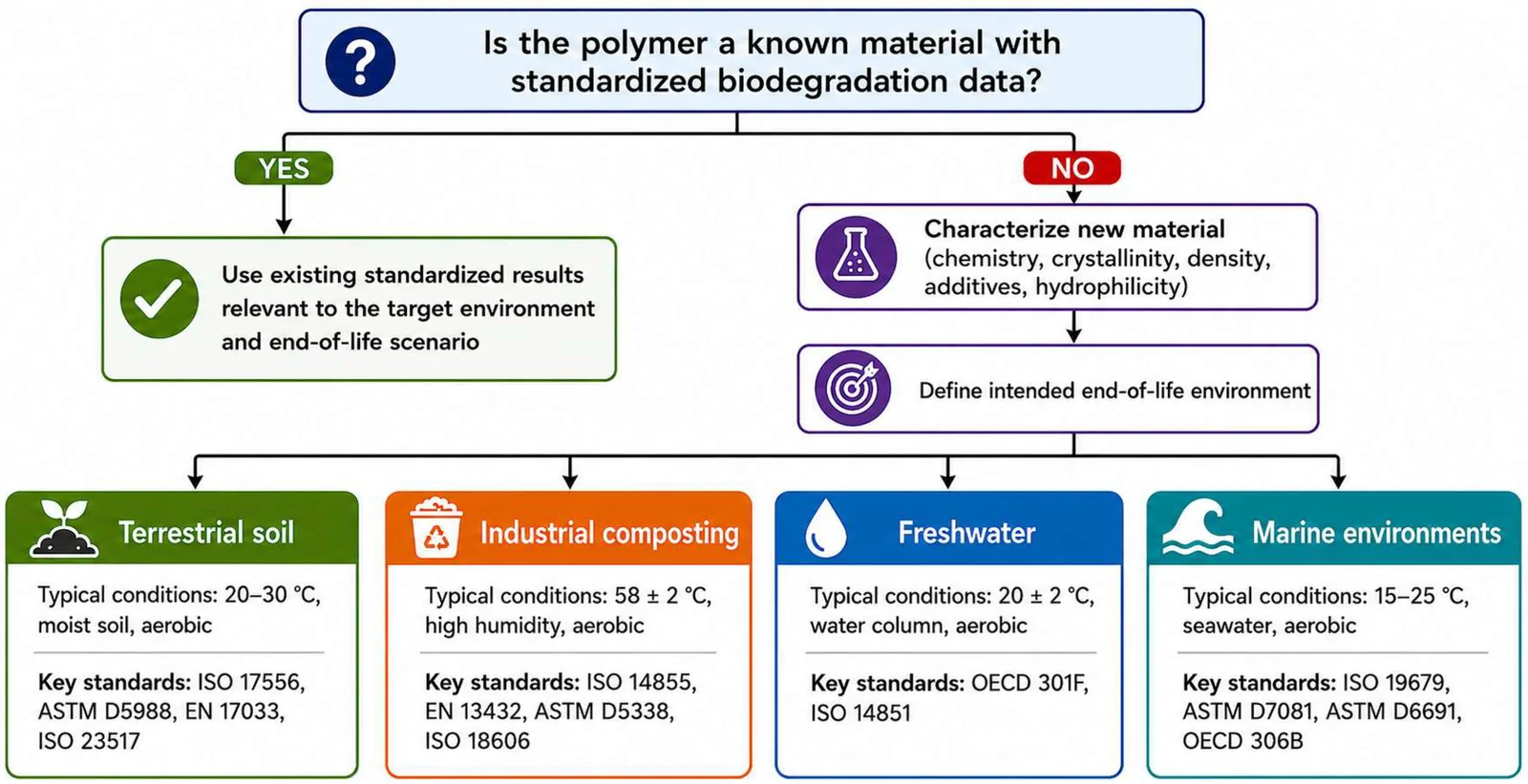
Decision framework for selecting standardized biodegradation test methods.

Plastic pollution in terrestrial environments has emerged as a critical concern, with soils increasingly recognized as major sinks for both conventional and biodegradable plastic materials [16]. The persistence of plastics in soil is driven by their inherent chemical recalcitrance, and their fragmentation under physical and biological weathering processes leads to the progressive generation of microplastics (MPs) and nanoplastics (NPs) [15]. These particles pose significant risks to soil ecosystems, altering the physical and chemical properties of the soil matrix, disrupting microbial community structure and function, and impairing the growth and physiology of soil-dwelling fauna and plants [15]. Figure 3 presents the Decision framework for selecting standardized biodegradation test methods.

Biodegradable plastics have been widely proposed as a strategy to mitigate plastic accumulation in soils. However, incomplete biodegradation under real environmental conditions may result in the persistent generation of biodegradable microplastic particles, complicating their environmental safety profile [39]. In this context, the availability of robust and standardized testing methods to evaluate plastic biodegradation in soil is essential. Standards such as ISO 17556, which quantifies ultimate aerobic biodegradability in soil through respirometric CO_2_ or O_2_ measurements, and ASTM D5988, which determines aerobic mineralization in soil under controlled conditions, represent key frameworks for this assessment [49,50]. Nevertheless, significant gaps remain in existing frameworks regarding ecologically relevant test conditions and threshold criteria. A detailed overview of the principal soil biodegradation standards and their key parameters is provided in Table 2.

The ISO 17556:2019 specifies a method for determining the ultimate aerobic biodegradability of plastic materials in soil, quantifying biodegradation either through oxygen demand measured respirometrically or through the amount of carbon dioxide evolved [22]. This dual-detection flexibility is one of the standard’s principal strengths, allowing laboratories to select the measurement approach best suited to their equipment and the polymer under investigation while still generating comparable mineralization endpoints. A key advantage of the method is its capacity to support a tiered, multi-purpose testing framework: beyond quantifying the extent of biodegradation, the same soil incubation system can be coupled with complementary assays such as nitrification-based ecotoxicity testing, enabling researchers to simultaneously assess biodegradability and potential ecological side effects within a single experimental setup, as demonstrated for biodegradable mulch film, which achieved full biodegradation without inhibiting soil nitrification potential (Table 3) [50].

The standard’s strict control of incubation variables and environmental conditions also allows for reproducible differentiation between highly biodegradable polymers and more recalcitrant ones; for instance, the application of the ISO 17556 procedure has clearly distinguished poly(hydroxybutyrate) as highly biodegradable in Mediterranean Alfisol soil, in contrast to poly(lactic acid) and low-density polyethylene, which showed poor biodegradability under the same conditions [53]. Overall, ISO 17556:2019 offers a robust, adaptable, and ecologically informative framework for soil biodegradability assessment.

A notable limitation concerns version consistency across literature: several studies applying the ISO 17556 method, such as that of Ardisson et al. [50], were conducted under the 2012 edition of the standard rather than its current 2019 revision, which complicates the direct comparability of results across the published record. Furthermore, biodegradation outcomes obtained under this standard appear to be strongly soil-dependent; Boluda et al. [53] demonstrated that material biodegradability assessed via ISO 17556 varied substantially with soil type and properties, such that results generated in one soil matrix (e.g., Mediterranean Alfisol) cannot necessarily be extrapolated to other environments. The short-term experimental design adopted in that study further constrains conclusions regarding long-term or field-realistic biodegradation behavior.

Taken together, these findings point to an absence of inter-laboratory reproducibility data and field validation studies for ISO 17556, raising questions about the broader external validity of laboratory-derived biodegradation rates obtained under this standard.

ASTM D5988 is a standard test method for determining the aerobic biodegradation of plastic materials in soil. It is based on measuring the cumulative amount of carbon dioxide produced when microorganisms break down the polymer in a controlled laboratory environment. A key strength of this standard is its demonstrated ability to differentiate the extent of biodegradation across structurally diverse polymers (Table 4).

Kishk et al. applied the ASTM D5988-18 protocol over a 180-day incubation period and clearly distinguished a co-polyester thermoplastic starch that reached near-complete mineralization within 28 days from linear low-density polyethylene, which showed minimal mineralization [54]. This confirmed the method’s ability to distinguish between polymers with different biodegradability levels. The standard lends itself well to mechanistic and environmental fate studies. For example, Chinaglia et al. used the ASTM D5988-18 method to quantify how soil water content affects mineralization kinetics. They established a predictable exponential relationship between water availability and disappearance time (DT50). This relationship allows biodegradation rates to be extrapolated across realistic environmental moisture conditions [49]. A limitation of ASTM D5988 emerges from its sensitivity to environmental moisture conditions: Chinaglia et al. [49] demonstrated that the mineralization rate under this method is strongly water-dependent, with disappearance time (DT50) varying from 42 to 190 days across different soil water contents, indicating that single-condition laboratory results may not reliably predict field biodegradation across variable moisture regimes.

EN 17033:2018 establishes requirements and test methods for biodegradable mulch films intended for agricultural and horticultural use. It defines a threshold for soil biodegradability that products must meet to qualify. A notable strength of this standard is its clear quantitative compliance benchmark: a 90% biodegradability threshold. This threshold provides an unambiguous pass/fail criterion that can be applied directly to experimental data. Chinaglia et al. demonstrated that a commercial biodegradable mulch film exceeded this threshold under adequate soil moisture conditions (13–19% soil water content). The film reached the threshold in under 90 days once moisture was restored. This illustrates the standard’s practical relevance for validating real agricultural products under realistic environmental variability. A recent study that applied the ASTM D5988-18 methodology and evaluated the results according to the EN 17033:2018 acceptance criterion demonstrated the critical influence of soil water content on polymer biodegradation. A commercial biodegradable plastic was incubated for ten months under aerobic soil conditions at three different soil water contents (SWC): 6%, 13%, and 19%. These corresponded to 25%, 55%, and 80% of the maximum water-holding capacity (WHC), respectively. Biodegradation exceeded the 90% mineralization threshold required by EN 17033 at 13% and 19% SWC. However, only partial degradation (<90%) was achieved at 6% SWC. This indicates that insufficient moisture significantly limits microbial activity and biodegradation. Water availability was also reflected in the half-life (DT_50_) values, which increased from 42 days at 19% SWC to 75 days at 13% SWC and 190 days at 6% SWC. These results clearly show that soil moisture is one of the most influential environmental factors that govern biodegradation under terrestrial conditions. They also emphasize the importance of maintaining an adequate water-holding capacity during standardized soil biodegradation testing.

Soil water content (SWC) is typically expressed as a percentage of the maximum water-holding capacity (WHC). This allows for the normalization of moisture conditions across different soil types. Since soils vary greatly in their ability to retain water, identical absolute moisture contents can indicate significantly different levels of water availability in sandy versus clay-rich soils. Therefore, expressing SWC relative to WHC provides a more meaningful basis for comparing biodegradation studies performed using different soil matrices. For instance, in Chinaglia et al.’s study [49], SWC values of 6%, 13%, and 19% corresponded to 25%, 55%, and 80% of the maximum WHC, respectively. Using the ASTM D5988-18 methodology and evaluating biodegradation performance against the EN 17033:2018 acceptance criterion, the authors demonstrated that the commercial biodegradable plastic achieved ≥90% biodegradation only at SWC values of 13% and 19%, whereas biodegradation remained below the acceptance threshold at an SWC value of 6%. These results clearly show that soil moisture is a critical factor in controlling microbial activity and polymer mineralization. Adequate water availability is necessary for biodegradable plastics to meet the compostability requirements specified by EN 17033:2018 under soil conditions.

ISO 23517:2021 (“Plastics-Soil Biodegradable Materials for Mulch Films for Use in Agriculture and Horticulture-Requirements and Test Methods Regarding Biodegradation, Ecotoxicity and Control of Constituents”) is the first international specification specifically designed to certify soil-biodegradable plastic products for agricultural use [52]. It evaluates three aspects: control of constituents (including heavy metals), biodegradation, requiring ≥90% conversion of carbon to CO_2_ within 24 months at room temperature via ISO 17556, and negative effects on terrestrial organisms [52]. Despite its relevance, very few independent scientific studies have explicitly adopted it, largely because it was only published in July 2021, leaving little time for the research community to incorporate it into experimental frameworks. Furthermore, ISO 23517 is inherently certification-oriented, meaning it tends to be adopted by industry for product labeling rather than by researchers exploring degradation mechanisms [57]. As a result, the standard currently functions more as a regulatory framework than as an active research tool, and independent experimental validation of its criteria across diverse soil types and climatic conditions remains largely absent from the scientific literature [12].

### 3.2. Standard Methodologies for Assessing Plastic Biodegradation in Compost

The most widely adopted standard methodologies for the biodegradation of plastics in compost are ISO 14855-1 and its gravimetric counterpart ISO 14855-2, along with the American equivalent ASTM D5338. All three determine ultimate aerobic biodegradability by measuring CO_2_ evolved from the test material under controlled thermophilic composting conditions [12]. For physical disintegration, ISO 16929 (pilot-scale) and ISO 20200 (lab-scale) were developed to determine the degree to which materials fragment during composting and are distinct from ultimate biodegradability tests [58].

The framework/specification standards are:

EN 13432, the European harmonized standard for compostable packaging, which integrates biodegradation (≥90% in 6 months at 58 ± 2 °C), disintegration (≥90% through 2 mm sieve after 12 weeks), ecotoxicity, and chemical characterization into a single framework.

ASTM D6400, the US equivalent, requires 90% disintegration (via ISO 16929 or ISO 20200) and ≥90% biodegradation (via D5338 or ISO 14855), among other ecotoxicity criteria. AS 5810, the Australian home composting standard with a maximum test period of 12 months, requiring at least 90% biodegradation at ambient temperature.

Table 5 offers a comparison of internationally recognized standards for assessing the biodegradability, disintegration, and compostability of plastic materials under industrial composting conditions.

ISO 14855-1 and ISO 14855-2 are the most widely recognized international standards for evaluating the aerobic biodegradability of plastics under controlled composting conditions. Both methods simulate industrial composting by maintaining thermophilic temperatures (58 ± 2 °C), adequate moisture content, and aerobic conditions that promote microbial activity and polymer mineralization. As summarized in Table 6, these standards form the basis of most compostability evaluations and are often referenced by certification programs such as EN 13432 and ASTM D6400.

The biodegradation assessment is based on the mineralization of the test material, which is quantified by measuring the amount of carbon dioxide (CO_2_) produced. The polymer is mixed with mature compost, which serves as the microbial inoculum, and incubated under controlled environmental conditions. The amount of CO_2_ generated during microbial metabolism is then compared to the amount produced by a positive reference material (typically microcrystalline cellulose) and blank compost controls. The degree of biodegradation is expressed as the percentage of the theoretical amount of CO_2_ released relative to the total organic carbon in the test material.

According to international compostability standards, biodegradable plastics are generally expected to convert at least 90% of their organic carbon into CO_2_ within 180 days to demonstrate extensive microbial mineralization rather than simple physical fragmentation [61,62]. Consequently, CO_2_ evolution has become the primary endpoint for evaluating the aerobic biodegradability of compostable plastics.

ISO 14855 standards are based on the same biodegradation principle; however, they differ in the analytical method used to quantify carbon dioxide. ISO 14855-1 uses continuous respirometric systems, which are typically based on infrared or volumetric detection. This allows for automated monitoring throughout the incubation period. In contrast, ISO 14855-2 uses a gravimetric approach, absorbing evolved CO_2_ in alkaline solutions such as sodium hydroxide or barium hydroxide and quantifying it by titration or gravimetric analysis. The gravimetric method requires simpler instrumentation and lower operating costs; however, the automated respirometric approach provides continuous data acquisition and is better suited for long-term kinetic studies.

Despite these differences in methodology, both standards provide comparable estimates of ultimate aerobic biodegradability because they are performed under identical composting conditions and use the same calculation principle based on theoretical CO_2_ production. Consequently, the choice between ISO 14855-1 and ISO 14855-2 is generally determined by laboratory infrastructure and analytical capabilities rather than by differences in the assessment of biodegradation itself [20,21].

Published literature from the last five years shows that these standards have been widely used to evaluate the biodegradation of polylactic acid (PLA), polyhydroxyalkanoates (PHAs), poly(butylene succinate) (PBS), poly(butylene adipate-co-terephthalate) (PBAT), and their blends and composites. Comparative analyses performed under ISO 14855 conditions have shown that biodegradation kinetics are strongly influenced by polymer chemistry, crystallinity, molecular weight, and blend composition. Meanwhile, compost maturity, moisture content, and microbial activity remain the principal environmental factors that govern mineralization rates. These studies, summirezed in Table 6, also highlight a significant limitation of industrial composting tests: excellent biodegradation performance under controlled laboratory conditions does not necessarily predict equivalent behavior in soil, freshwater, or marine environments. This emphasizes the need for environment-specific assessment methodologies.

ASTM D5338 has become a cornerstone standard for evaluating the aerobic biodegradation of plastic materials under controlled composting conditions. This standard provides a reproducible methodology for quantifying biodegradation through measurement of carbon dioxide evolution, enabling researchers to compare the performance of different biopolymer formulations under consistent conditions.

The fundamental principle of ASTM D5338 involves measuring the conversion of carbon in the test material to carbon dioxide (CO_2_) under aerobic conditions. Test specimens are mixed with mature compost that serves as both a microbial inoculum and a substrate matrix. The mixture is maintained at thermophilic temperatures, typically 58 ± 2 °C, which represents the optimal temperature range for thermophilic microorganisms commonly found in industrial composting facilities [32]. The evolved CO_2_ is captured and quantified, usually through titration methods using barium hydroxide or potassium hydroxide solutions or through gas chromatography techniques [69].

The standard specifies several critical parameters to ensure test validity and reproducibility. The compost medium must meet specific quality criteria, including appropriate pH (typically 7–9) and moisture content (50–55%), oxygen concentration (>6%), and demonstrate microbial activity as measured by respiration rate [70]. Test materials are typically ground or cut into small particles to increase surface area and facilitate microbial colonization. The material-to-compost ratio is standardized, commonly at 1:6 (*w*/*w* sample to dry solids of compost), to prevent substrate overloading that could inhibit microbial activity [28,71].

A key feature of ASTM D5338 is the use of positive and negative controls. Microcrystalline cellulose typically serves as positive control, as it is a readily biodegradable natural polymer that should achieve >70% mineralization within the test period. Polyethylene or other recalcitrant plastics serve as negative controls, which should show minimal or no biodegradation [70]. The biodegradation percentage is calculated based on the theoretical maximum CO_2_ that could be produced from complete mineralization of the carbon content in the test material.

The standard typically requires test durations ranging from 45 to 180 days; however, some studies extend beyond this period to achieve plateau biodegradation levels [72]. Materials are generally considered compostable if they achieve at least 60–90% biodegradation within 180 days, depending on the specific certification requirements (e.g., ASTM D6400 for compostable plastics) [73]. The represemtatove studies found are summarized in Table 7. 

ISO 16929:2021 and ISO 20200:2015/2023 are physical fragmentation standards; they quantify the degree of disintegration, defined as the percentage of sample mass that passes through a 2 mm sieve after a controlled composting cycle. As explicitly stated in the ISO 16929:2021 normative text itself, “this test method cannot be used to determine the aerobic biodegradability of a test material” [59].

ISO 20200 was developed specifically to assess disintegration in a laboratory-scale composting environment, in contrast to ISO 16929 and ISO 14045, which are designed for pilot-scale composting tests. In both standards, no gas-phase measurement of CO_2_ or O_2_ is performed; consequently, neither standard provides evidence of actual biochemical mineralization of the polymer into CO_2_, water, and biomass. This contrasts sharply with standards such as ISO 14855 (controlled composting respirometry), ISO 17556 (soil respirometry), and ASTM D5338/D5988, which quantify ultimate aerobic biodegradability through the measurement of evolved CO_2_ relative to the theoretical carbon content of the material. ASTM D6400 and ISO 17088 were developed as more comprehensive specifications that encompass both biodegradability and disintegration requirements, alongside ecotoxicological safety assessment [74].

Despite this distinction, disintegration measurement is not a mere procedural formality; it is mechanistically indispensable as an indicator of the first obligatory stage of the biodegradation process.

From a regulatory and certification standpoint, ISO 20200 is extremely useful as a screening method for indicating potential biodegradation prior to committing to a resource-intensive full biodegradation study, such as those required by ISO 14855 or ASTM D5338. ISO 16929 could represent the most adequate alternative for characterizing biopolymers in a less idealized and more realistic composting environment, as its pilot-scale setup is much more representative of real microbial populations than laboratory-scale configurations [12].

Table 8 and Table 9 show the results of studies conducted after 2020 that used ISO 16929:2021 and ISO 20200:2015 to quantify the degree of disintegration of several biopolymers.

ASTM D6400 is a standard specification for labeling plastics designed to be aerobically composted in municipal or industrial facilities. Beyond the core assessment of biodegradability measured through CO_2_ evolution and disintegration testing, the standard encompasses a broader set of analyzes, including elemental analysis, phytotoxicity (plant germination), and mesh filtration of residual particles, requiring that a material achieve ≥90% conversion of organic carbon to CO_2_ within 180 days, disintegrate to less than 10% residue after 12 weeks, and produce compost that supports ≥90% plant germination relative to a blank control. These criteria are evaluated exclusively under thermophilic aerobic conditions (~58 °C) representative of large-scale industrial composting, which is precisely where the standard’s usefulness ends. Passing ASTM D6400 confirms industrial compostability, but it says nothing about what happens to a material in a garden soil, a river, or the ocean. Nomadolo et al. (2022) [32] showed that biopolymers performing well under composting conditions can exhibit negligible degradation in soil, where temperatures are lower and microbial communities differ fundamentally. Even within the composting scenario itself, Chong et al. (2024) [78] found that biopolymer blends that disintegrated readily in laboratory tests retained over 93% of their mass when placed in a real industrial composting plant, exposing a significant gap between controlled protocols and real-world outcomes. The standard also does not address anaerobic degradation, which is relevant for landfill disposal and home composting, for which no internationally harmonized standard currently exists [79]. As Pires et al. [12] pointed out, ASTM D6400 is best understood as a compostability specification rather than a universal biodegradability tool: complementary standards such as ISO 17556 or ASTM D5988 for soil.

AS 5810:2010 is an Australian standard developed to evaluate whether biodegradable plastics are suitable for home composting conditions, with criteria focused on biodegradation, disintegration, and ecotoxicity under relatively low and variable temperatures compared with industrial composting systems [63]. Unlike industrial compostability standards, AS 5810 specifically addresses decentralized household composting environments, which are generally less controlled and more difficult to reproduce experimentally [68]. Despite its relevance for sustainable packaging research, relatively few peer-reviewed studies directly apply AS 5810, as most biodegradability studies continue to rely on internationally dominant standards such as EN 13432 or ASTM-based protocols. The relatively small number of studies using AS 5810 may be due to the difficulty of reproducing realistic home composting conditions, where factors such as temperature, moisture levels, and microbial activity naturally vary and can make results harder to compare across different studies.

EN 13432 is the European standard that specifies requirements and procedures for determining the compostability and anaerobic treatability of packaging and packaging materials [25]. The EN 13432 standard establishes four main criteria for compostable packaging materials. The first is biodegradation; the material must achieve at least 90% biodegradation within 180 days under controlled composting conditions. Biodegradation is measured as the conversion of organic carbon to CO_2_ through aerobic mineralization, typically assessed using respirometric methods based on ISO 14855-1 or ISO 14855-2 standards [80]. The second criterion is disintegration: at least 90% of the material must pass through a 2 mm sieve after 12 weeks (84 days) of composting. This requirement ensures that the material physically breaks down into small fragments that do not negatively impact compost quality [74]. For this criterion, ISO 16929 is the standard that is most used. The third criterion is ecotoxicity: the resulting compost must not exhibit adverse effects on plant growth. This is assessed through plant growth tests comparing compost containing the test material with control compost [81]; OECD 208 defines the steps to assess ecotoxicity. And the last one is the heavy metal content: the material must meet specified limits for heavy metals (lead, cadmium, mercury, chromium, etc.) to ensure the safety of the final compost product [81]. The standards used for these criteria are ISO 14853 and EN 14047. EN 13432 has been criticized for relying on static, tightly controlled conditions that do not reflect the dynamic biological processes of real industrial or natural environments [81]. Materials certified under this standard have shown markedly lower disintegration rates in actual composting facilities compared to laboratory results [78]. 

### 3.3. Standard Methodologies for Assessing Plastic Biodegradation in Aquatic Systems

Plastic pollution has emerged as one of the most pervasive environmental threats of the 21st century, with aquatic systems bearing a disproportionate burden of its consequences. The persistence of conventional plastics in water bodies drives their progressive fragmentation into microplastics (MPs) and nanoplastics (NPs), particles of increasing ecotoxicological concern due to their ability to accumulate in animal and human tissues [82]. 

Marine debris, where plastic accounts for the vast majority of recorded encounters with wildlife, has been shown to affect hundreds of aquatic species, with a significant proportion already classified as threatened [43]. Biodegradable plastics have been proposed as an alternative, yet their degradation behavior in aquatic environments remains poorly understood, and incomplete breakdown can result in the persistent generation of biodegradable microplastic particles [38,44].

In this context, the establishment of reliable and standardized methods to assess plastic biodegradation specifically in aquatic matrices is critical, as existing frameworks present significant gaps that hinder accurate environmental assessment.

Several international standards have been developed to evaluate plastic biodegradation in aquatic systems, including ISO 14851 and ISO 14852, which assess ultimate aerobic biodegradability in aqueous media through oxygen demand and carbon dioxide evolution measurements. A detailed overview of these and other related standards is provided in Table 10.

Beyond the standards outlined in Table 10, three further methods are used in a complementary capacity. ISO 15314:2018 is a physical marine exposure protocol used as a conditioning pre-step prior to separate analytical assessment [12]. ISO 22766:2020 measures macroscopic fragmentation under real field conditions and is explicitly classified by ISO as a disintegration test, insufficient to substantiate biodegradability claims. ISO 62:2008 characterizes water absorption capacity, a material property indicative of hydrolytic susceptibility, but incorporates no biological or microbiological component [91]. Collectively, these methods serve as complementary characterization tools rather than standalone evidence of aquatic biodegradability.

#### 3.3.1. Freshwater

ISO 14851:2019 is one of the primary international standards used to evaluate the aerobic biodegradability of plastics in water. This standard determines biodegradation by measuring the biochemical oxygen demand (BOD) produced by microbial metabolism in a closed respirometer containing an aqueous inoculum, usually activated sludge from wastewater treatment plants. By simulating aerobic freshwater conditions in a controlled laboratory setting, the standard provides a reproducible approach for evaluating the environmental fate of biodegradable plastics and water-soluble polymers. Due to its robustness and reproducibility, ISO 14851:2019 is often referenced in studies examining polymer biodegradability, environmental risk assessment, and the regulatory evaluation of biodegradable materials. However, as shown in Table 11, relatively few studies have applied the standard directly to generate primary biodegradation data. Instead, many publications reference ISO 14851 conceptually or use it as a benchmark when comparing alternative biodegradability assessment methodologies [12]. The limited number of experimental studies also reflects the growing preference for complementary CO_2_-based methods (e.g., ISO 14852) and marine-specific protocols, especially for polymers intended for aquatic environments.

Poli et al. [92] demonstrated that ISO 14851:2019 presents critical limitations for assessing bioplastic biodegradability in freshwater, including the absence of a defined biodegradability threshold, excessive flexibility in process parameter selection, high variability attributed to inoculum quality, and the omission of complementary characterization analyzes and ecotoxicological testing, collectively hindering the reliable and reproducible classification of polymeric materials as biodegradable.

ISO 14852:2021 is one of the primary international standards used to evaluate the complete aerobic biodegradation of plastic materials in water. Unlike ISO 14851, which uses biochemical oxygen demand (BOD) to quantify biodegradation, ISO 14852 determines the extent of mineralization by measuring the carbon dioxide (CO_2_) produced during microbial metabolism. Therefore, the method provides a direct, quantitative assessment of the conversion of polymer-derived carbon into CO_2_ under controlled, aerobic conditions, reflecting the ultimate biodegradation of the test material [84].

A significant change was introduced in the 2021 edition of ISO 14852 concerning the selection of microbial inoculums. While earlier versions of the standard permitted the use of soil and mature compost in addition to activated sludge, the current edition restricts the inoculum to activated sludge obtained exclusively from a well-operated wastewater treatment plant. This change was made to improve the reproducibility and standardization of biodegradation testing by reducing variability from different inoculum sources. However, this change also has important implications for interpreting and comparing published studies. Results generated according to previous editions of ISO 14852 may not be directly comparable to those obtained under the revised protocol because soil, compost, and activated sludge differ substantially in terms of microbial diversity, enzymatic activity, and biodegradation potential. Therefore, when comparing biodegradation data across different studies, careful consideration should be given to the version of the standard employed.

EN ISO 14852:2021 does not specify a biodegradability threshold for definitive material classification, and variations in inoculum sources among laboratories can introduce significant variability, thereby limiting the reproducibility and cross-study comparability of biodegradation data.

The results summarized in Table 12 demonstrate the marked influence of polymer chemistry on biodegradation in aqueous media under ISO 14852:2018 conditions. Among the polymers investigated, polyhydroxyalkanoates (PHAs) exhibited the highest biodegradation, with PHBV (87.4 ± 7.5%) and PHB (83.0 ± 1.6%) showing extensive mineralization within 117 days. Poly(ε-caprolactone) (PCL) also displayed substantial biodegradation, reaching 77.6 ± 2.4% after 177 days, confirming its susceptibility to enzymatic hydrolysis in activated sludge systems. In contrast, PBS, PBAT, and PLA exhibited negligible biodegradation during the same incubation period, indicating that these polymers hydrolyze much more slowly under mesophilic aqueous conditions than under industrial composting conditions. These findings emphasize that polymer performance is highly dependent on the testing environment and demonstrate that results obtained under composting standards such as ISO 14855 cannot be directly extrapolated to freshwater biodegradation assessed according to ISO 14852. The study further highlights the superior biodegradability of microbial polyesters compared with synthetic aliphatic polyesters under activated sludge conditions, reflecting differences in polymer structure, enzyme specificity, and microbial adaptation [94].

While aerobic biodegradation tests (such as ISO 14851 and ISO 14852) are commonly used to assess plastic biodegradability in oxygenated water columns, ISO 14853:2016 offers a complementary approach for evaluating biodegradation under anaerobic conditions. This is particularly important because the biodegradation behavior of plastics can differ significantly between aerobic and anaerobic environments due to differences in microbial communities, metabolic pathways, and degradation kinetics [95]. This literature analysis reveals a significant gap: while the standard is frequently referenced in review papers and methodological discussions, very few studies report actual experimental data using ISO 14853:2016 for freshwater biodegradability assessment. Only a limited number of experimental studies have directly applied ISO 14853:2016.

Under mesophilic anaerobic digestion (36 ± 1 °C, municipal sludge inoculum, 77 days), PHB and PHBV show the highest biodegradation, with values above 80%, indicating strong susceptibility to anaerobic mineralization. In contrast, PLA remains essentially recalcitrant, with only a few percent mineralization, while PBS, PCL, and PBAT display intermediate degradation levels spanning roughly 20–45%. A PLA/PCL (60/40) blend exhibits less than 2% biodegradation, highlighting the significant impact of copolymer and blend composition on anaerobic degradability. One of the most significant challenges in applying ISO 14853:2016 is the variability in anaerobic inocula used for testing. The biodegradation potential of an inoculum depends on its microbial community composition, which can vary significantly depending on the source (e.g., anaerobic digester sludge, freshwater sediment, wastewater treatment plant sludge) [90]. 

To improve the reproducibility and environmental relevance of ISO 14853:2016 testing for freshwater applications, efforts should be made to develop standardized freshwater sediment inocula with characterized microbial communities, establish protocols for inoculum preparation and quality control specific to freshwater environments, and investigate the biodegradation adaptation potential (BAP) of different freshwater inocula.

#### 3.3.2. Seawater

ISO 18830:2016 is one of the primary international standards used to evaluate the aerobic biodegradability of plastic materials in seawater. This method measures biodegradation by determining the biochemical oxygen demand (BOD) resulting from the microbial degradation of test materials under controlled marine conditions. Test specimens are incubated in sealed respirometric vessels containing natural or artificial seawater and sandy marine sediment to simulate the sublittoral benthic environment. Decreases in dissolved oxygen are continuously monitored or periodically measured and expressed as a percentage of theoretical oxygen demand (ThOD). This provides a quantitative estimate of polymer mineralization.

Despite its relevance, a survey of recent literature indicates that ISO 18830:2016 is rarely used as the primary methodology for marine biodegradability assessment. Most recent studies instead employ ISO 19679:2020, which evaluates the same environmental compartment and determines biodegradation through carbon dioxide evolution rather than oxygen consumption. The increasing preference for ISO 19679 is primarily attributed to its direct measurement of carbon mineralization, reduced interference from nitrification processes, and compatibility with widely available CO_2_ respirometric systems.

ISO 19679:2020 represents the most frequently applied international standard for evaluating the ultimate aerobic biodegradability of plastic materials at the seawater–sediment interface. Like ISO 18830, it reproduces benthic marine conditions using natural seawater and marine sediment but quantifies biodegradation by measuring evolved carbon dioxide (CO_2_) rather than oxygen consumption [26]. Because both standards simulate identical environmental conditions, their results are generally considered directly comparable, with the principal difference lying in the analytical endpoint employed [12]. Representative studies applying ISO 19679:2020 are summarized in Table 13.

As shown in Table 13, studies indicate that PBS-based fishing fibers exhibit only moderate biodegradation after 180 days, with mineralization values ranging from 22 to 27%. Interestingly, incorporating 10 wt.% PHA into PBS resulted in only a modest increase in biodegradation compared to pure PBS, suggesting that small changes in polymer composition are not enough to significantly speed up degradation in marine environments. Similarly, previously used fishing fibers exhibited slightly lower biodegradation than unused materials, potentially due to changes in polymer morphology or surface properties resulting from prolonged environmental exposure [97]. These findings illustrate the inherently slow biodegradation kinetics characteristic of marine environments, even for polymers designed to be biodegradable.

Although ISO 19679:2020 has become the preferred methodology for laboratory-based marine biodegradability assessment, several important limitations remain. Similar to ISO 18830, the standard applies only to materials that sink and remain at the seawater–sediment interface. This excludes low-density polymers that float on the water’s surface. This is a significant limitation because floating plastics make up a large part of marine litter and are exposed to different degradation mechanisms, including ultraviolet radiation, photooxidation, wave-induced abrasion, and biofouling. Furthermore, biodegradation data generated at the sediment–water interface cannot be directly extrapolated to other ecologically relevant marine compartments, such as the pelagic water column, coastal surface waters, or deeply buried sediments. Therefore, certification based solely on ISO 19679 should not be interpreted as evidence of complete marine biodegradability, but rather as an assessment specific to one well-defined environmental compartment. This limitation underscores the necessity of additional methodologies that can evaluate polymer degradation across diverse marine habitats under real environmental conditions [12,26].

Similarly to ISO 18830:2016, ISO 19679:2020 is restricted to materials with a density exceeding that of seawater, systematically excluding positively low-density polymers from its scope. This represents a critical methodological gap, as floating plastics constitute a substantial proportion of marine debris and undergo distinct degradation pathways driven by UV exposure and wave action. Furthermore, by confining its evaluative framework exclusively to the sediment–water interface, the standard produces biodegradation data that cannot be extrapolated to other ecologically significant marine compartments, such as the pelagic zone or fully buried sediment. This limitation is particularly consequential from a regulatory standpoint, as materials certified under ISO 19679 may exhibit markedly different, and potentially negligible, degradation behavior across the full spectrum of marine environments, thereby risking the misrepresentation of biodegradability claims.

ISO 23977-1:2020 specifies a method to determine the ultimate aerobic biodegradability of plastic materials in the marine water column. It measures the evolution of carbon dioxide (CO_2_) under pelagic conditions. Unlike ISO 19679:2020, which evaluates biodegradation at the seawater–sediment interface, ISO 23977-1 simulates the open marine water column. This makes it particularly suitable for floating or neutrally buoyant polymeric materials that remain suspended in seawater. The method uses natural coastal seawater as the microbial inoculum and measures biodegradation by quantifying the mineralization of polymer-derived carbon into CO_2_ under controlled aerobic conditions.

ISO 23977-1:2020 requires that incubation shall be maintained at a constant mesophilic temperature, preferably between 15 °C and 25 °C and not exceeding 28 °C (±1 °C); any deviation from this range must be justified and reported [86]. This mesophilic requirement reflects temperate coastal seawater conditions rather than tropical or subtropical settings. Consequently, ISO 23977-1 complements benthic marine standards by providing a methodology designed to evaluate polymers more likely to remain in the pelagic zone than to settle at the sediment–water interface.

Despite its relevance, ISO 23977-1:2020 has seen limited application in the scientific literature. A systematic review of publications from after 2021 found few experimental studies that directly applied this methodology to assess marine biodegradability. One of the few available investigations was reported by Vikman et al., who evaluated the biodegradation of kraft lignin under aerobic seawater conditions using the ISO 23977-1 protocol. The material exhibited 13.1% ± 1.1% biodegradation after 120 days when incubated in Baltic Sea seawater at 22 °C, indicating relatively limited mineralization under pelagic marine conditions. Although lignin is a naturally occurring biopolymer, its complex aromatic structure and high recalcitrance likely contributed to the slow biodegradation observed during the test period [97].

The limited number of studies using ISO 23977-1 reflects the standard’s recent introduction and the practical challenges of its implementation. Continuous monitoring of CO_2_ evolution requires specialized respirometric equipment and careful control of experimental conditions throughout long incubation periods. This makes the methodology more technically demanding than several alternative marine biodegradation tests. Additionally, since pelagic marine biodegradation generally occurs much more slowly than degradation under composting or soil conditions, experiments often necessitate extended incubation periods before meaningful levels of mineralization can be detected.

Nevertheless, ISO 23977-1 fills an important methodological gap by providing a standardized framework for assessing the biodegradation of floating plastics under environmentally relevant marine conditions. As concerns about plastic accumulation in the open ocean grow, the broader application of this standard will be essential to generate comparable data sets and improve the environmental assessment of emerging biodegradable polymers intended for marine applications.

ISO 23977-2:2020, the companion standard to ISO 23977-1, specifies a method for determining the aerobic biodegradability of plastic materials exposed to seawater. This method involves measuring oxygen demand in a closed respirometer. It provides an alternative measurement endpoint (oxygen consumption) for the same pelagic exposure conditions. Like ISO 23977-1:2020, ISO 23977-2:2020 does not appear to be among the standards commonly used by researchers to assess polymer biodegradability. This can be attributed to several methodological constraints associated with the standard. The protocol permits testing durations of up to two years to achieve a significant level of biodegradation. The oxygen consumed during the testing period, which may extend up to two years, is compared to the theoretical oxygen demand to determine the degree of biodegradation of the plastic. Such extended timeframes substantially limit the technique’s throughput, increase associated costs, and heighten the likelihood of accidental data loss during prolonged incubation periods. This issue has been noted in critiques of similarly long marine biodegradation standards [98].

Extended test durations of up to two years and the high operational and equipment costs associated with long-term closed-respirometer incubations likely explain why researchers have favored shorter, modified, or alternative protocols, such as the 28-day method developed by López-Ibáñez and Beiras (2022) [99].

ISO 22403:2020 is an important step toward harmonizing marine biodegradability assessments. It establishes common requirements and reference materials for evaluating the biodegradation of plastic materials in marine environments. A major strength of the standard is its provision of a unified framework that improves the comparability of results generated by different laboratories and testing approaches [88]. However, ISO 22403:2020 cannot be used as a standalone method because it does not specify a unique procedure to measure biodegradation. Instead, the standard relies on complementary test methods, such as ISO 18830, ISO 19679, and ISO 22404, to generate actual biodegradation data. In practice, ISO 22403 functions more as a guideline for interpreting and validating biodegradation results than as a laboratory protocol. While this approach offers flexibility and allows testing under different marine scenarios, it also introduces variability between studies, making direct comparisons of biodegradation performance more challenging [100].

ASTM D6691 specifies a standard test method for determining the aerobic biodegradation of plastic materials in the marine environment using natural seawater with native microbiota [43]. The standard emphasizes the use of natural, unmodified seawater to maintain ecological relevance [43]. The standard underlies major certification schemes, including TÜV Austria’s “OK biodegradable MARINE” label, supporting industry-recognized marine-biodegradability claims. Its relatively short incubation window (10–90 days, extendable to 180 days for certification) makes it more practical than multi-year seawater/sediment alternatives such as ISO 18830 or ISO 19679. Selected examples of polymers whose biodegradability was assessed using ASTM D6691 in studies published after 2020 are presented in Table 14.

ASTM D6691 carries several recognized limitations that affect its environmental relevance and reproducibility. The enriched marine solution specified in the standard contains ammonia and nitrate concentrations several orders of magnitude higher than those found in natural seawater, conditions that bear little resemblance to the nutrient-poor (oligotrophic) state of most ocean waters [102].

The fixed incubation temperature of 30 ± 2 °C is similarly difficult to reconcile with real marine settings, where temperature drops considerably with depth and can shift the biodegradation process in ways the standard does not capture [103]. A further weakness lies in the test’s lack of systematic validation: parameters such as aeration adequacy, bioreactor dimensions, and sample particle size are not tightly constrained, and insufficient oxygen supply or unrepresentative particle sizing can both distort respirometric outcomes [103], Because few studies have rigorously assessed the standard’s performance, some researchers have turned to modified versions of D6691, adjusting nutrient levels, reactor size, and aeration frequency in an effort to improve reliability and bring laboratory conditions closer to those of real pelagic coastal environments [104]. Such adaptations point to a persistent gap between standardized testing protocols and the ecological realities they are meant to approximate.

ASTM D7991:2015 is specifically designed for materials that sink and become buried in marine sediments, representing a distinct environmental compartment from the water column or sediment–water interface [96]. Sandy marine sediment contains diverse microbial communities, including bacteria, fungi, and archaea, that may differ from water column communities, providing a distinct biodegradation environment [90]. 

An extended literature search identified only a single study employing ASTM D7991 for the assessment of biopolymer biodegradability in marine sediment. Meereboer et al. [29] applied this standard to evaluate the marine biodegradation of PHBV and its biocomposites with natural fillers, representing, to date, the sole reported application of this specific test method within the available literature on biopolymer degradation. The results of this study are presented in Table 15 below.

ASTM D7991 presents several methodological constraints that limit its routine application in biodegradability research. The mandated test duration of up to 24 months is impractical for typical research and product-development timelines and substantially increases experimental costs relative to shorter standards [102].The standard is further restricted to sandy sediments, whereas natural marine substrates are highly heterogeneous, with vast regions of the seafloor covered by fine-grained sediments such as mud, which may exhibit markedly different degradation kinetics [103]. Its exclusively aerobic design also fails to represent fine-grained, organic-rich sediments, which are frequently anoxic below the surface layer [24], while testing at a fixed temperature of 25 °C does not account for thermal variability across marine environments. Finally, the periodic moisture-replenishment protocol inadequately reproduces natural porewater dynamics, since tidal pumping and wave action drive advective porewater exchange in intertidal and subtidal sediments, a process not captured under static laboratory conditions.

There is no single standardized methodology that can be applied universally to all biodegradable polymers and environmental scenarios. Although they require longer testing periods and specialized equipment, respirometric methods based on CO_2_ evolution or O_2_ consumption generally provide the most reliable evidence of ultimate biodegradation. In contrast, disintegration and mass-loss tests are simpler and faster, but they primarily assess physical deterioration rather than complete mineralization. Therefore, the selection of an appropriate standard depends on the polymer family, intended application, target environment, measured endpoint, required level of reproducibility and regulatory purpose. Consequently, standardized methodologies should be regarded as complementary rather than interchangeable tools for assessing polymer biodegradability.

## 4. Conclusions and Future Perspectives

The increasing demand for sustainable polymeric materials has led to a substantial increase in the need for reliable, internationally harmonized methodologies to evaluate biodegradability under environmentally relevant conditions. As demonstrated throughout this review, numerous standardized protocols are currently available for assessing polymer biodegradation in soil, compost, freshwater, and marine environments. Each standard has been developed to replicate a specific environmental setting and apply different experimental conditions, inocula, and analytical endpoints. Consequently, biodegradation data generated using different standards should not be considered directly comparable without careful consideration of the testing methodology. Published literature from the last five years highlights the growing adoption of internationally recognized standards, such as ISO 17556, ISO 14855, ASTM D5338, ISO 14851, ISO 14852, ISO 19679, and ASTM D6691. This reflects an increasing effort toward harmonized biodegradability assessment. However, the analysis also reveals substantial differences in experimental design among studies, including variations in inoculum source, environmental conditions, specimen preparation, and reporting of biodegradation endpoints. These inconsistencies limit inter-study comparability and complicate the interpretation of polymer biodegradability across different environmental compartments. A recurring observation throughout the reviewed literature is that polymer biodegradation is governed not only by polymer chemistry but also by environmental conditions. Temperature, moisture, oxygen availability, microbial diversity, crystallinity, molecular weight, morphology, and specimen geometry can all influence degradation kinetics. Therefore, excellent biodegradation performance under industrial composting conditions should not be interpreted as evidence of equivalent behavior in soil, freshwater, or marine environments. The selection of an appropriate testing standard should instead reflect the material’s intended end-of-life scenario. This review also shows that most standardized methodologies evaluate ultimate mineralization under controlled laboratory conditions, whereas considerably fewer studies investigate polymer behavior under environmentally realistic field conditions. Some environmental compartments, particularly freshwater and marine sediments, remain underrepresented in the literature, and several recently published standards have been applied in only a limited number of experimental studies. Broader validation of existing methodologies across a wider range of polymer classes and environmental matrices is therefore required. Field studies have also demonstrated that materials showing substantial degradation under controlled or industrial composting conditions may behave very differently in home composting or other real environments, further emphasizing the limitations of extrapolating laboratory results directly to environmental performance [104]. Future research should consequently focus on improving the environmental relevance, reproducibility, and harmonization of biodegradation testing. Greater effort is needed to standardize inoculum preparation, characterize microbial communities, establish appropriate controls and reference materials, and adopt unified reporting criteria that facilitate comparison among independent studies. Laboratory-based biodegradation assessments should increasingly be complemented by field validation studies to better predict polymer performance under real environmental conditions. Particular attention should also be given to distinguishing physical disintegration or surface modification from depolymerization and ultimate mineralization, since these processes do not provide equivalent evidence of biodegradation. Polymer biodegradability cannot be treated as an intrinsic, fixed material property; rather, it emerges from the interaction of polymer chemistry, physical microstructure, and the biotic and abiotic conditions of a specific environment. The evidence gathered across ISO, ASTM, EN, and OECD frameworks confirms that crystallinity, molecular weight, and monomer composition govern the initial susceptibility of a polymer to abiotic hydrolysis and enzymatic attack, while temperature, moisture, oxygen availability, and microbial community composition determine whether depolymerization proceeds to substantial mineralization within a realistic timeframe. Aliphatic polyesters such as PLA, PHB/PHBV, PBAT, and PBS, together with polysaccharide-based materials such as starch and cellulose, illustrate this dependency clearly: their ester and glycosidic linkages can hydrolyze readily under the thermophilic, high-moisture conditions specified in ISO 14855-1, ISO 14855-2, ASTM D5338, and EN 13432, whereas the same materials may degrade considerably more slowly or incompletely under the mesophilic soil conditions represented by ISO 17556 and ISO 23517. Polyamides and polyesteramides (PEAs), whose amide linkages confer greater hydrolytic resistance, further demonstrate that chemical class alone cannot predict degradation outcome. Performance must therefore be established empirically for each polymer–environment pairing, and certification under one standardized set of conditions should not be interpreted as evidence of equivalent performance in another environment. Recent advances in microbial biotechnology further broaden the perspective of polymer degradation research. Microorganisms capable of transforming highly recalcitrant synthetic polymers under controlled conditions have been isolated and characterized, while progress in enzyme discovery, genomics, proteomics, and genetic engineering offers opportunities to enhance polymer depolymerization and biotransformation [105,106]. In particular, recombinant microbial strains and engineered enzymes may improve the biological conversion of polymers that exhibit little or no measurable degradation under conventional environmental conditions. However, degradation achieved by using selected or engineered biological systems under optimized biotechnological conditions should not automatically be interpreted as evidence of biodegradability in natural environments. Future standardization efforts should therefore distinguish clearly between environmentally occurring biodegradation, controlled biotechnological depolymerization or biotransformation, and ultimate mineralization. Such frameworks should also define appropriate requirements for microbial characterization, reference materials, controls, analytical endpoints, and confirmation of polymer carbon conversion to mineralization products [107]. Future testing frameworks should additionally consider technologically important polymer classes that are currently less represented in standardized biodegradation assessment. Natural rubber and, particularly, vulcanized rubber constitute relevant examples because of the environmental significance and persistence of tyre-derived particulate waste. Although the degradation of natural rubber by specific microorganisms and rubber-cleaving enzymes has been investigated, vulcanized rubber is substantially more resistant because of its highly cross-linked structure and complex additive composition. Recent studies have nevertheless demonstrated biologically mediated modifications of vulcanized rubber by fungal strains, with degradation-related effects associated with oxidative enzymes such as laccases and manganese peroxidases [108]. These findings highlight the potential for future biotechnological treatment strategies while simultaneously emphasizing the need for harmonized methodologies capable of distinguishing surface modification, depolymerization, biotransformation, and ultimate biodegradation in complex polymer systems. A clear hierarchy of degradation performance emerges from the comparative analysis of the reviewed studies, with industrial composting generally providing more favorable conditions for degradation than soil, freshwater, and marine environments. This gradient reflects differences in temperature, moisture, oxygen availability, and microbial activity across environmental matrices, but also the disproportionate attention historically given to compost and soil systems relative to aquatic and marine settings. Freshwater protocols, such as ISO 14851 and ISO 14852, and marine methods, including ISO 19679, ISO 23977, ASTM D6691, and ASTM D7991, remain comparatively less represented in experimental studies, with greater variability in inoculum characteristics and more limited consensus on evaluation criteria. Closing this gap is important for producing environmentally relevant assessments of polymer waste, particularly for polyesteramides, polyester blends, and other materials proposed for applications where freshwater or marine exposure may occur. For practitioners and policymakers, methodology selection should therefore be dictated by the intended end-of-life scenario rather than by the convenience or availability of a single generic protocol. Materials designed for industrial composting, agricultural soil incorporation, wastewater exposure, or applications associated with possible freshwater or marine release each require distinct testing pathways aligned with the actual disposal or leakage route. Future work should prioritize harmonization of experimental conditions and reporting criteria across standards bodies, expansion and validation of protocols for underrepresented environments and polymer classes, integration of emerging microbial and enzymatic technologies into appropriately defined testing frameworks, and systematic pairing of laboratory results with field validation. Such developments are essential for building a more predictive, reproducible, and environmentally credible framework for evaluating polymer biodegradability.

## Figures and Tables

**Table 1:** Biodegradation in different environments.

Parameter	Soil	Compost	Freshwater	Seawater
Typical temperature	10–30 °C	50–60 °C (industrial compost)	4–25 °C	2–25 °C
Moisture	Moderate, variable	High (50–60%)	High	High
Oxygen availability	Generally aerobic	Aerobic	Aerobic (variable)	Aerobic (often lower in sediments)
Dominant microorganisms	Bacteria, fungi, actinomycetes	Thermophilic bacteria, fungi, and actinomycetes	Aquatic bacteria, fungi, algae	Marine bacteria, fungi, algae
Main degradation mechanisms	Hydrolysis, enzymatic depolymerization, microbial assimilation	Accelerated hydrolysis and enzymatic degradation	Hydrolysis, biofilm- mediated degradation	Slow hydrolysis, biofilm formation, enzymatic degradation
Environmental characteristics	Heterogeneous matrix; variable pH and nutrient availability	High microbial activity and elevated temperature	Low nutrient concentration; fluctuating physicochemical conditions	Low temperature, high salinity, nutrient limitation
Relative biodegradation rate	Moderate	Very fast	Slow– moderate	Slowest
Typical degradation products	CO_2_, H_2_O, biomass	CO_2_, H_2_O, biomass	CO_2_, H_2_O, biomass	CO_2_, H_2_O (aerobic); CH_4_ under anaerobic sediment conditions
Representative standards	ISO 17556; ASTM D5988	ISO 14855-1/-2; ASTM D5338; EN 13432; ASTM D6400	ISO 14851; ISO 14852; OECD 301	ISO 18830; ISO 19679; ASTM D6691
Main limitations	High variability between soil types	Simulates Industrial rather than natural conditions	Limited standardization and long test durations	Slow degradation, high environmental variability, limited harmonized standards

**Table 2:** Comparison of the internationally recognized standards for the assessment of biodegradation in soil, highlighting the measured biodegradation parameter, test duration, environmental conditions, specimen requirements, and reference materials.

Standard	Parameter Measured (O_2_/CO_2_)	Standard Duration	Temperature	Material Formulation	Moisture Holding Capacity	Reference Material	Reference
ISO 17556:2019	O_2_ or CO_2_ (both accepted)	180 days standard; extendable if degradation is still active	20–28 °C	Powder preferred (≤250 µm); soil sieved <2 mm	40–60% WHC; pH 6–8	Microcrystalline cellulose, ashless cellulose filter, or PHB	[22]
ASTM D5988-18	CO_2_ only	Until plateau; typically ≤180 days—no fixed endpoint	22–28 °C	Small, uniform pieces (film, pellets, powder); no strict size limit	50–70% WHC (D2980) or 80–100% (D425); pH 6–8	Cellulose or starch; ≥60% conversion at plateau required for test validity	[24]
EN 17033:2018	CO_2_ (delegates to ISO 17556)	Up to 720 days to reach ≥90% pass	Ambient; preferably 25 °C	Powder or film form (per ISO 17556)	40–60% WHC (per ISO 17556)	Microcrystalline cellulose (per ISO 17556)	[51]
ISO 23517:2021	CO_2_ (delegates) to ISO 17556)	Up to 720 days to reach ≥90% pass	Ambient; preferably 25 °C (per ISO 17556)	Powder preferred (≤250 µm, per ISO 17556)	40–60% WHC (per ISO 17556)	Microcrystalline cellulose (per ISO 17556)	[52]

**Table 3:** Representative studies reporting the biodegradation of polymeric materials in soil according to ISO 17556:2019. Biodegradation percentages, test duration, experimental conditions, and polymer formulations are summarized.

Polymer	Biodegradation [%]	Duration	Conditions	Reference
PHB	Without cow manure, 8.76% With cow manure, 0.52%	20 days	Mediterranean Alfisol	[53]
PLA	Without cow manure: 0.73% With cow manure −1.10% (negative value, explained by the control soil consuming more oxygen than the sample)	20 days	Mediterranean Alfisol	[49]
LDPE	Without cow manure, 0.82% With cow manure, 0.31%	20 days	Mediterranean Alfisol	[49]
PBSA + PHEF formulation	62.4% in 90 days → 90.5% in 361 days	361 days	Standard soil, 25 ± 2 °C	[54]
PBAT	9.2% at 58 °C/2.3% at 25 °C	33 weeks	Uncontaminated soil	[55]
PLA	6.1% at 58 °C/1.7% at 25 °C	33 weeks	Uncontaminated soil	[55]

**Table 4:** Summary of representative studies investigating the aerobic biodegradation of polymeric materials in soil under ASTM D5988 conditions. The table compares biodegradation percentages, incubation periods, testing conditions, and compliance with EN 17033:2018 requirements where reported.

Polymer	Biodegradation [%]	Duration of Test	Conditions	Reference
TPS2 (Co-polyester thermoplastic starch)	~100%	28 days	25 °C, compost–soil matrix, CO_2_ evolution, aerobic	[54]
Starch (native)	71.10%	180 days	25 °C, compost–soil matrix, CO_2_ evolution, aerobic	[54]
LLDPE (control)	~0%	180 days	25 °C, compost–soil matrix, CO_2_ evolution, aerobic	[50]
PLA/PBAT-g-MAH/Bamboo bio composites	Reported as % Mineralization (early-time, ≤3–4 weeks)	≤4 weeks	~21 °C, soil–compost, titrimetric CO_2_ capture, aerobic	[55]
PHA	100%	90 days	Soil, aerobic, under standard ASTM D5988 conditions	[56]
PCL	100%	270 days	Soil, aerobic, standard ASTM D5988 conditions	[52]
PHA/PLA blend	99%	176 days	Soil, aerobic, ASTM D5988-12 protocol	[52]
PHBHx + Joncryl chain extender (5%)	22%	340 days	Soil, aerobic; chain extender reduced biodegradation from 70% to 22%	[52]

**Table 5:** Comparison of internationally recognized standards for assessing the biodegradability, disintegration, and compostability of plastic materials under industrial composting conditions. This table summarizes the biodegradation parameters measured, the incubation time, the temperature, the specimen requirements, the moisture conditions, and the reference materials specified by each standard.

Standard	Parameter Measured	Standard Duration	Temperature	Material Formulation	Moisture Content	Reference Material	Reference
ISO 14855-1	Ultimate aerobic biodegradability (% CO_2_ evolved vs. ThCO_2_)	45–180 days	58 ± 2 °C	Test material: typically <25 mm; inoculum: mature compost	~50–60% water content of compost inoculum	Microcrystalline cellulose (MCC, <20 µm, TLC grade)	[20]
ISO 14855-2	Ultimate aerobic biodegradability via gravimetric CO_2_ measurement	45–180 days	58 ± 2 °C	Test material: typically <25 mm; inoculum: mature compost + inert sea sand	~50–60%	Microcrystalline cellulose (MCC, TLC grade)	[21]
ASTM D5338	Rate and degree of aerobic biodegradation (% CO_2_ evolved vs. ThCO_2_)	45–180 days	58 ± 2 °C	Test material: typically <25 mm; inoculum: mature compost	~50–60%	Microcrystalline cellulose (MCC)	[23]
ISO 16929	Degree of physical disintegration (% dry weight passing 2 mm sieve)	84 days (12 weeks)	45–75 °C (variable thermophilic range)	Input biowaste: max 50 mm; samples: films 10 × 10 cm, rigid items 5 × 5 cm	>50% water content (maintained throughout)	Blank compost control; cellulose paper strips to assess compost activity	[59]
ISO 20200	Degree of physical disintegration (% dry weight passing 2 mm sieve)	45–90 days	58 °C	Synthetic waste matrix: 95% < 1 cm; test material pre-cut to specified size	~55% (controlled and maintained)	Cellulose filter paper or blank compost control (without test material)	[60]
EN 13432	Biodegradation + disintegration + ecotoxicity + chemical characterization	180 days (biodeg.); 84 days (disintegration)	58 ± 2 °C (via ISO 14855)	Not independently defined; references ISO 16929 and ISO 20200	>50% (references ISO 14855 and ISO 16929)	Microcrystalline cellulose (via ISO 14855 for biodeg. sub-test)	[61]
ASTM D6400	Biodegradation + disintegration + ecotoxicity + heavy metals (labeling spec.)	Min. 90 days (series); biodeg. up to 180 days	>50 °C thermophilic (via ASTM D5338)	Not independently defined; follows D5338/ISO 16929/ISO 20200	Not independently specified; follows ASTM D5338 and ISO 16929/20200 conditions	Microcrystalline cellulose (via ASTM D5338 for biodeg. sub-test)	[62]
AS 5810	Biodegradation + disintegration + ecotoxicity + earthworm toxicity (home conditions)	Up to 365 days (12 months)	20–30 °C (ambient home) composting)	Similar to EN 13432; home composting inoculum; Particle size is not strictly controlled	Variable/ ambient; not strictly controlled—reflects realistic home conditions	Cellulose/MCC (equivalent to EN 13432 requirements)	[63]

**Table 6:** Summary of representative studies investigating the ultimate aerobic biodegradability of biodegradable polymers under controlled composting conditions according to ISO 14855-1 and ISO 14855-2. Reported biodegradation values, incubation periods, testing conditions, and polymer formulations are compared.

Polymer	Biodegradation %	Duration of Test	Conditions of Test	ISO Standard	Reference
TPS/PLA blend	84.7 ± 0.3%	120 days	Simulated natural env. conditions	ISO 14855-2:2007	[64]
TPS (2% MG)	82.4 ± 0.4%	120 days	Simulated natural env. conditions	ISO 14855-2:2007	[62]
TPS (3% MG)	82.0 ± 0.1%	120 days	Simulated natural env. conditions	ISO 14855-2:2007	[62]
TPS (6% MG)	81.0 ± 0.3%	120 days	Simulated natural env. conditions	ISO 14855-2:2007	[62]
TPSEG	66.7 ± 2.4%	120 days	Simulated natural env. conditions	ISO 14855-2:2007	[62]
TPSE	62.9 ± 1.0%	120 days	Simulated natural env. conditions	ISO 14855-2:2007	[62]
PLA-1OLA films	98.6%	Not specified	58 ± 2 °C, 50 ± 5% moisture, >6% O_2_	ISO 14855-1:2012	[65]
PHBV-5OLA films	85.5%	Not specified	58 ± 2 °C, 50 ± 5% moisture, >6% O_2_	ISO 14855-1:2012	[63]
Multilayer PLA/PHBV	73.1%	Not specified	58 ± 2 °C, 50 ± 5% moisture, >6% O_2_	ISO 14855-1:2012	[63]
PLA-based materials	47.46–98.34%	6 months	Controlled aerobic composting	ISO 14855-1:2012	[66]
PBS-based materials	34.15–80.36%	6 months	Controlled aerobic composting	ISO 14855-1:2012	[64]
PLA/MC2192 films	~12%	28 days	Controlled thermophilic composting	ISO 14855-1	[27]
PLA/ATBC films (as produced)	26.0%	70 days	Controlled thermophilic composting	ISO 14855-1	[22]
PLA/ATBC films (aged)	19.0%	70 days	Controlled thermophilic composting	ISO 14855-1	[22]
PLA/MC2178 films (aged)	34.0%	70 days	Controlled thermophilic composting	ISO 14855-1	[22]
PHBV film (pure)	100.0%	40 days	Aerobic biodegradability, 45 days of incubation	ISO 14855	[67]
PHBV double-coated papers	23.0%	45 days	Aerobic biodegradability test	ISO 14855	[67]
PHBV (pure)	100.0%	90 days	Industrial composting	ISO 14855-1	[68]
PHBV/TC composite	99.0%	90 days	Industrial composting	ISO 14855-1	[66]
PHBV/WF composite	97.0%	90 days	Industrial composting	ISO 14855-1	[66]

**Table 7:** Representative studies evaluating the ultimate aerobic biodegradability of polymeric materials under controlled composting conditions using ASTM D5338 and related compostability specifications (ASTM D6400, where applicable). The table summarizes biodegradation percentages, test duration, experimental conditions, and polymer formulations reported in the literature.

Polymer	Biodegradation %	Duration of the Test	Conditions of the Test	Reference
CD/PEG-PBAT blend (60% PBAT)	41%	108 days	Simulated aerobic composting, 58 ± 2 °C, 50–55% RH	[28]
CD/PEG-PLA blend (40% PLA)	107%	108 days	Simulated aerobic composting, 58 ± 2 °C, 50–55% RH	[23]
Neat PLA	91%	108 days	Simulated aerobic composting, 58 ± 2 °C, 50–55% RH	[23]
Neat PBAT	69%	108 days	Simulated aerobic composting, 58 ± 2 °C, 50–55% RH	[23]
Neat CD (cellulose diacetate)	100%	108 days	Simulated aerobic composting, 58 ± 2 °C, 50–55% RH	[23]
Microcrystalline cellulose (MCC)	107%	108 days	Simulated aerobic composting, 58 ± 2 °C, 50–55% RH	[23]
Sepia melanin	37.0%	98 days	ASTM D5338, thermophilic (58 °C), CO_2_ measurement	[70]
Cellulose (positive control)	97 ± 6%	98 days	ASTM D5338, thermophilic (58 °C)	[68]
Cellulose (positive control)	71.2 ± 0.2%	97 days	ASTM D5338, mesophilic (25 °C)	[68]
Polyethylene (negative control)	0	98 days	ASTM D5338, thermophilic (58 °C)	[68]
PBAT-PBS blend	~82%	120 days	ASTM D5338 & D6400, industrial composting, (58 °C)	[32]
PBAT-PLA blend	~90%	120 days	ASTM D5338 & D6400, industrial composting, (58 °C)	[27]
PBAT-PBS blend	72.0%	200 days	ASTM D5338 & D6400, home composting, (28 °C)	[27]
PBAT-PLA blend	50.0%	200 days	ASTM D5338 & D6400, home composting, (28 °C)	[27]
Cellulose (positive control)	70.0%	45 days	ASTM D5338 & D6400, industrial composting, (58 °C)	[27]

**Table 8:** Representative studies evaluating the disintegration of biodegradable polymeric materials under controlled composting conditions according to ISO 16929:2021. The table summarizes the degree of disintegration, incubation period, composting conditions, and polymer formulations reported in the literature.

Polymer	Disintegration [%]	Duration	Test Conditions	Reference
PLA standard rigid packaging/films	>90% disintegration (pass criterion met); dependent on temperature and form factor	12 weeks	Pilot-scale aerobic composting; thermophilic (>55 °C ≥ 4 weeks, >50 °C for ≥4 weeks); synthetic biowaste; ISO 16929 (disintegration component of ASTM D6400)	[40]
Starch-based biopolymer carrier bags	95% disintegration by week 12	12 weeks	Large-scale composting; 37.5 t manure/wood + biopolymer bags; peak T ~70 °C; aerobic; ISO 16929-aligned pilot conditions	[75]
PBAT/PLA compostable mulch film	Passes disintegration (<10% residue at 12 weeks); biodegradation 60–90% at 180 days	12 weeks (disintegration); 180 days (biodegradation)	Disintegration per ISO 16929; biodegradation per ASTM D5338; thermophilic; 58 °C	[76]

**Table 9:** Representative studies evaluating the disintegration of biodegradable polymeric materials under laboratory-scale simulated composting conditions according to ISO 20200:2015. The table summarizes the degree of disintegration, incubation period, composting conditions, and polymer formulations reported in the literature, along with complementary biodegradation results determined according to ISO 14855 where available.

Polymer	Disintegration [%]	Duration	Test Conditions	Reference
PLA-1OLA (PLA plasticized with oleic acid, 10 wt.%)— monolayer film	91.13% disintegration; 98.59% biodegradation (CO_2_ evolution)	45 days (disintegration); up to 90 days (biodegradation)	Lab-scale simulated composting; 58 ± 2 °C; 50 ± 5% moisture; >6% O_2_; ISO 20200:2016 + ISO 14855-1	[65]
PHBV-5OLA (PHBV plasticized with oleic acid, 5 wt.%)—monolayer film	89.93% disintegration	45–90 days	Lab-scale simulated composting; 58 ± 2 °C; 50 ± 5% moisture; ISO 20200:2016	[63]
PLA-1OLA/PHBV-5OLA multilayer film (co-extruded bilayer)	79.18% disintegration	45–90 days	Lab-scale simulated composting; 58 ± 2 °C; 50 ± 5% moisture; ISO 20200:2016	[63]
PLA—flexible films/certified waste bags (EN 13432)	Partial disintegration; visible plastic remaining after 90 days for rigid items	90 days	Lab-scale simulated composting; 58 °C; 55% moisture; ISO 20200; commercial products	[74]
PBAT/starch—flexible compostable bags (commercial)	Disintegration started on day 35; ≥90% of one product by day 90	90 days	Lab-scale simulated composting; 58 °C; 55% moisture; ISO 20200	[72]
PHA—flexible waste bags (commercial)	Disintegration started on day 38; visible plastic remained at 90 days	90 days	Lab-scale simulated composting; 58 °C; 55% moisture; ISO 20200	[72]
PLA/PBS fibers (multiple cross-section geometries)	PLA: several fully degraded samples; PBS: all samples intact after 45 days	45 days	Lab-scale ISO 20200 disintegration; thermophilic composting; samples pre-aged by UV/hydrolysis	[72]
Bioderived polyurethane–polyester foam composites	~25.4 ± 3.6% mass loss (disintegration) in 45 days	45 days	Lab-scale simulated composting; ISO 20200 conditions; 58 °C; aerobic	[77]
PBS—Flexible packaging (BPE-SP-PBS)	45.4% remaining at wk. 4; 10.9% at wk. 8; 0.3% at wk.12 (~99.7% disintegration)	12 weeks	Lab-scale ISO 20200; 58 °C; 55% moisture; synthetic biowaste (40% sawdust, 30% rabbit feed, 10% compost, 5% sucrose)	[78]

**Table 10:** Comparison of the principal international standards used for assessing the biodegradability of polymeric materials in freshwater and marine environments. The table summarizes the biodegradation endpoint, standard incubation period, temperature, specimen preparation, test medium, and reference materials specified by each methodology.

Standard	Parameter Measured	Duration (Standard)	Temperature	Material Formulation	Medium	Reference Material	Reference
Fresh water/Aqueous Medium							
ISO 14851:2019	Ultimate aerobic biodegradability oxygen demand (BOD) in a closed respirometer	Up to 6 months (≤182 days)	25 ± 1 °C	Powder preferred; also, films, pieces, or shaped articles; free of additives (e.g., plasticizers)	Fully aqueous medium with activated sludge inoculum (freshwater/wastewater)	Microcrystalline cellulose (MCC); validity: ≥60% biodegradation for reference material	[83]
ISO 14852:2021	Ultimate aerobic biodegradability evolved CO_2_ in an aqueous medium.	Up to 6 months (≤182 days)	20–25 °C (±1 °C)	Powder preferred; also, films, pieces, or shaped articles; free of additives	Fully aqueous medium with activated sludge inoculum (freshwater/wastewater)	MCC, unbleached cellulose filter, or PHB; validity: ≥60% biodegradation at end of test	[84]
ISO 14853:2016	Ultimate anaerobic biodegradability biogas (CH_4_ + CO_2_) production	Up to 90 days	35 ± 2 °C	Granular or finely divided material; water-soluble polymers or plastics with additives allowed	Aqueous anaerobic slurry—digested sludge (1–3 g/L total solids)	PHB, microcrystalline cellulose, or PEG 400; validity: ≥70% biodegradation of reference	[85]
	Marine/Sea Water						
ISO 18830:2016	Aerobic biodegradability O_2_ demand in a closed respirometer at the seawater/sandy sediment interface	Up to 24 months	15–25 °C (±2 °C) recommended: 20 °C	Non-floating films or flat specimens; materials that settle at the seawater/sediment interface	Natural or synthetic seawater + sandy marine sediment (sublittoral benthic zone simulation)	MCC or poly(hydroxybutyrate) (PHB); validity: ≥60% biodegradation for reference after 180 days	[25]
ISO 19679:2020	Aerobic biodegradability evolved CO_2_ at the seawater/sediment interface	Up to 24 months	15–25 °C (±2 °C)	Non-floating films or flat specimens; materials that sink to the seawater/sediment interface	Natural or synthetic seawater + marine sediment (sublittoral benthic zone simulation)	Amorphous cellulose filter; validity: ≥60% biodegradation for reference and CO_2_ ≤ 20% of total	[26]
ISO 23977-1:2020	Aerobic biodegradability in the seawater column (pelagic/suspended sediment) evolved CO_2_	Normally, up to 1 year; extendable to 2 years if significant biodegradation is ongoing	15–25 °C	Films, powders, or shaped articles; pelagic (seawater only) or suspended sediment (seawater + 0.1–1 g/L sediment) test variant	Natural coastal seawater inoculum; no additional activated sludge, marine microorganism community	MCC or ashless cellulose filters; validity: ≥60% biodegradation of reference after 180 days	[86]
ISO 23977-2:2020	Aerobic biodegradability in the seawater column (pelagic/suspended sediment) O_2_ demand in a closed respirometer	Normally up to 1 year; extendable to 2 years if significant biodegradation is ongoing	20–25 °C	Films, powders, or shaped articles; ≥100 mg/L test substance or 170 mg/L ThOD; pelagic or suspended sediment variant	Natural coastal seawater inoculum; 300 mL reactor with 90 mL seawater ± sediment (0.1–1 g/L)	MCC or ashless cellulose filters; validity: ≥60% biodegradation of reference after 180 days	[87]
ISO 22403:2020	Intrinsic biodegradability in marine water CO_2_ evolution (aerobic, mesophilic)	Up to 2 years	25 ± 2 °C	Films or particles; test materials exposed to marine inoculum under mesophilic aerobic conditions	Marine seawater inoculum (open ocean or coastal); fully aqueous	MCC; Test material: ≥90% mineralization (vs. cellulose reference) within 2 years; virgin PE negative control required; underlying method’s own validity criteria also apply	[88]
ASTM D6691 (latest version: 2024)	Aerobic biodegradability in the marine environment: CO_2_ evolution	10–90 days (extendable to 180 days)	30 ± 2 °C	Powders, films, or shaped articles; without plasticizers or additives preferred; high surface area recommended	Defined microbial consortium or natural seawater inoculum with added inorganic nutrients	Cellulose powder or paper; validity: ≥70% biodegradation for reference after 6 months	[89]
ASTM D7991:2015	Aerobic biodegradability: CO_2_ evolution and mass loss in buried sandy marine sediment (lab conditions)	Up to 24 months	Ambient laboratory conditions (~20–25 °C)	Flat specimens or films buried in sandy marine sediments under controlled laboratory conditions	Sandy marine sediment: specimens buried and kept under controlled humidity/seawater saturation	MCC; validity criteria similar to ISO 18830/19679	[90]

**Table 11:** Representative studies investigating the ultimate aerobic biodegradability of polymeric materials in freshwater environments using the ISO 14851:2019 methodology. Reported biodegradation values are presented together with the corresponding incubation period, testing conditions, and polymer formulations.

Polymer	Biodegradation [%]	Duration of the Test	Conditions of the Test	Reference
Cellulose diacetate	72.30%	118 days	Temperature: 21 °C; Inoculum source: activated sludge from Belgian wastewater treatment plants	[92]
PLA	44.04%	60 days	Temperature: 20 °C; Inoculum source: Activated sludge from the recirculation well of the secondary settling tank of the Ca’ Nordio wastewater treatment plant (Padova, Italy)	[93]
PLA	23.08%	60 days	Temperature: 20 °C; Inoculum source: Activated sludge from the activated sludge tank of the Ca’ Nordio wastewater treatment plant (Padova, Italy)	[93]

**Table 12:** Representative studies evaluating the ultimate aerobic biodegradability of polymeric materials in aqueous media according to ISO 14852:2018. The table summarizes the reported biodegradation percentages, incubation periods, experimental conditions, and polymer formulations under controlled freshwater conditions.

Polymer	Biodegradation (%)	Duration of Test	Test Conditions	Standard Version	Reference
PHB	83.0% ± 1.6%	117 days	Temperature: 25 ± 1 °C; Activated sludge from the Valladolid sewage treatment plant (Spain)	ISO 14852:2018	[94]
PHBV	87.4% ± 7.5%	117 days	Temperature: 25 ± 1 °C; Activated sludge from the Valladolid sewage treatment plant (Spain)	ISO 14852:2018	[93]
PCL	77.6% ± 2.4%	177 days	Temperature: 25 ± 1 °C; Activated sludge from the Valladolid sewage treatment plant (Spain)	ISO 14852:2018	[93]
PBS	Negligible (~0%)	117 days	Temperature: 25 ± 1 °C; Activated sludge from the Valladolid sewage treatment plant (Spain)	ISO 14852:2018	[93]
PBAT	Negligible (~0%)	117 days	Temperature: 25 ± 1 °C; Activated sludge from the Valladolid sewage treatment plant (Spain)	ISO 14852:2018	[93]
PLA	Negligible (~0%)	117 days	Temperature: 25 ± 1 °C; Activated sludge from the Valladolid sewage treatment plant (Spain)	ISO 14852:2018	[93]

**Table 13:** Representative studies evaluating the ultimate aerobic biodegradability of polymeric materials at the seawater–sediment interface according to ISO 19679:2020. The table summarizes the reported biodegradation percentages, incubation periods, experimental conditions, and polymer formulations.

Polymer	Biodegradation [%]	Duration of the Test	Conditions of the Test	Reference
PBS-fishing fiber	27.30%	180 days	Seawater/sediment interface; natural marine sediment inoculum; Seawater and sediment sourced from Daebu Island, Ansan, Korea	[43]
PBS—used fishing fiber	22.10%	180 days	Seawater/sediment interface; natural marine sediment inoculum; Seawater and sediment sourced from Daebu Island, Ansan, Korea; fiber pre-used in the marine fisheries environment	[96]
PBS/PHA (9:1) blend fiber	25.90%	180 days	Seawater/sediment interface; natural marine sediment inoculum; Seawater and sediment sourced from Daebu Island, Ansan, Korea; 10 wt.% PHA added to PBS fishing gear fiber	[97]

**Table 14:** Representative studies evaluating the aerobic biodegradability of polymeric materials in marine environments according to ASTM D6691. The table summarizes the reported biodegradation percentages, incubation periods, seawater testing conditions, and polymer formulations published in recent literature.

Polymer/Material	Biodegradation (%)	Test Duration	Test Conditions	Reference
PHB, biosynthesized	12.8% (natural seawater); 86% (synthetic seawater)	28 days	Natural and synthetic Thailand seawater, aerobic respirometric CO_2_ evolution	[95]
PHBVV, biosynthesized	61.2% (natural seawater); 96.5% (synthetic seawater)	28 days	Natural and synthetic Thailand seawater, aerobic respirometric CO_2_ evolution	[94]
Polylactic acid (PLA) nonwoven fiber/textile	0.20%	28 days	Bioreactor test, natural seawater inoculum from the open sea (Belgium) enriched with inorganic nutrients (0.05 g/L NH_4_Cl, 0.1 g/L KH_2_PO_4_), 30 ± 2 °C, duplicate samples, third-party lab (Organic Waste Systems, Belgium); cellulose reference reached 85.2% (±3.8%)	[101]
Lyocell (CLY) regenerated cellulose nonwoven fiber	76.0% (± 2.7%)	28 days	Bioreactor test, natural seawater inoculum (Belgium), 30 ± 2 °C, duplicate samples; cellulose reference reached 85.2% (±3.8%) in same run	[101]
Modal (CMD) regenerated cellulose nonwoven fiber	81.4% (± 2.0%)	28 days	Bioreactor test, natural seawater inoculum (Belgium), 30 ± 2 °C, duplicate samples; cellulose reference reached 85.2% (±3.8%) in same run	[102]
Viscose (CV) regenerated cellulose nonwoven fiber	82.2% (± 1.2%)	28 days	Bioreactor test, natural seawater inoculum (Belgium), 30 ± 2 °C, duplicate samples; cellulose reference reached 85.2% (±3.8%) in same run	[102]
Organic virgin cotton (OCO) natural cellulose nonwoven fiber	81.2%	28 days	Bioreactor test, natural seawater inoculum (Belgium), 30 ± 2 °C, duplicate samples; cellulose reference reached 85.2% (±3.8%) in same run	[102]

**Table 15:** Representative study evaluating the aerobic biodegradability of PHBV-based materials buried in marine sandy sediments according to ASTM D7991:2015. The table summarizes the reported biodegradation percentages, incubation periods, experimental conditions, and material formulations.

Material	Biodegradation [%]	Test Duration	Test Conditions	Reference
Virgin PHBV (powder)	89.42%	450 days	25 ± 2 °C; sediment: marine water 250 g:150 g; powder form; marine sediment & water from Ripley’s Aquarium, Toronto	[29]
PHBV/Miscanthus biocomposite (85/15 wt.%)	+15% biodegradation extent relative to virgin PHBV (same time frame)	450 days	25 ± 2 °C; powder form; 15 wt.% lignocellulosic Miscanthus fibre filler	[24]
PHBV/Miscanthus biocomposite (75/25 wt.%)	~97% (and +25% relative to virgin PHBV)	412 days	25 ± 2 °C; powder form; 25 wt.% lignocellulosic Miscanthus fibre filler	[24]
PHBV/DDGS biocomposite (85/15 wt.%)	+17% biodegradation extent relative to virgin PHBV (by day 295)	295 days	25 ± 2 °C; powder form; 15 wt.% proteinaceous distillers’ dried grains with soluble (DDGS) filler	[24]
PHBV/DDGS biocomposite (75/25 wt.%)	+40% biodegradation extent relative to virgin PHBV (by day 295)	295 days	25 ± 2 °C; powder form; 25 wt.% proteinaceous DDGS filler	[24]

## Data Availability

No new data were created or analyzed in this study. Data sharing is not applicable to this article.

## Abbreviations

The following abbreviations are used in this manuscript:

TPS	Thermoplastic starch
GLU	Soy protein with wheat gluten
LLDPE	Linear low-density polyethylene
LDPE	Low-density polyethylene
